# When to rely on maternal effects and when on phenotypic plasticity?

**DOI:** 10.1111/evo.12635

**Published:** 2015-04-10

**Authors:** Bram Kuijper, Rebecca B. Hoyle

**Affiliations:** ^1^ CoMPLEX, Centre for Mathematics and Physics in the Life Sciences and Experimental BiologyUniversity College LondonLondonUnited Kingdom; ^2^ Department of GeneticsEvolution and Environment, University, College LondonLondonUnited Kingdom; ^3^ Environment and Sustainability InstituteUniversity of Exeter, Penryn, CampusTremoughUnited Kingdom; ^4^ Department of MathematicsUniversity of SurreyGuildford GU2 7XHUnited Kingdom; ^5^ Mathematical SciencesUniversity of SouthamptonHighfieldSouthampton SO17 1BJUnited Kingdom

**Keywords:** environmental change, epigenetics, indirect genetic effect, maternal inheritance, nongenetic effect, phenotypic plasticity

## Abstract

Existing insight suggests that maternal effects have a substantial impact on evolution, yet these predictions assume that maternal effects themselves are evolutionarily constant. Hence, it is poorly understood how natural selection shapes maternal effects in different ecological circumstances. To overcome this, the current study derives an evolutionary model of maternal effects in a quantitative genetics context. In constant environments, we show that maternal effects evolve to slight negative values that result in a reduction of the phenotypic variance (canalization). By contrast, in populations experiencing abrupt change, maternal effects transiently evolve to positive values for many generations, facilitating the transmission of beneficial maternal phenotypes to offspring. In periodically fluctuating environments, maternal effects evolve according to the autocorrelation between maternal and offspring environments, favoring positive maternal effects when change is slow, and negative maternal effects when change is rapid. Generally, the strongest maternal effects occur for traits that experience very strong selection and for which plasticity is severely constrained. By contrast, for traits experiencing weak selection, phenotypic plasticity enhances the evolutionary scope of maternal effects, although maternal effects attain much smaller values throughout. As weak selection is common, finding substantial maternal influences on offspring phenotypes may be more challenging than anticipated.

Central to an organism's development is how it integrates cues about its genes and the environment to produce a phenotype that matches prevailing selective conditions (Müller, 2007; Carroll, 2008; Leimar, 2009; Beldade et al., 2011). It is now increasingly recognized that in addition to genetic and environmental factors, maternal effects also have a crucial influence on phenotypic development (Mousseau and Fox, 1998; Räsänen and Kruuk, 2007; Badyaev, 2008; Maestripieri and Mateo, 2009). Indeed, the transmission of maternal factors such as hormones (Groothuis and Schwabl, 2008), nutrients (Wells, 2003), antibodies (Boulinier and Staszewski, 2008), small RNAs (Liebers et al., 2014), or heritable epimutations (Li et al., 2008) affects offspring phenotypes and fitness in numerous taxa (e.g., Agrawal et al., 1999; Storm and Lima, 2010; McGhee et al., 2012; Holeski et al., 2012). Determining how maternal effects affect organismal adaptation is therefore a key part of the contemporary research agenda in evolutionary biology (Danchin et al., 2011; Uller, 2012).

Theoretical studies have shown that maternal effects, here defined as the causal influence of the maternal phenotype on the offspring's phenotype (Wolf and Wade, 2009), have multifaceted evolutionary consequences (Uller, 2008; Day and Bonduriansky, 2011). For example, maternal effects can change the response to selection (Kirkpatrick and Lande, 1989; Räsänen and Kruuk, 2007; Hoyle and Ezard, 2012; Ezard et al., 2014; Townley and Ezard, 2013) and play a crucial role in parent–offspring coadaptation (e.g., Wolf and Brodie, 1998; Kölliker, 2005). Although these studies provide important predictions about consequences of maternal effects, they typically assume that maternal effects are evolutionarily constant parameters. It is currently poorly understood how evolution shapes the evolution of maternal effects themselves across different ecological contexts. Here, we therefore use an evolutionary model of maternal effects to address this question.

Maternal effects reflect a form of phenotypic plasticity that spans generations (i.e., transgenerational plasticity; Uller, 2008). This raises the question of whether maternal effects evolve in similar contexts to within‐generational plasticity, which is selectively favored when (1) environments are heterogeneous (Berrigan and Scheiner, 2004), (2) costs of plasticity are low (Auld et al., 2010), and (3) environmental cues are informative (Reed et al., 2010). Indeed, variable environments and limited costs have also been associated with the evolution of maternal effects (Groothuis et al., 2005; Marshall and Uller, 2007; Uller, 2008). However, similarities between within‐generational plasticity and maternal effects break down when considering environmental cues: whereas models of within‐generational plasticity typically assume that cues directly reflect the state of the environment (e.g., Berrigan and Scheiner, 2004), models of maternal effects consider that offspring rely on the maternal phenotype as the source of environmental information (Uller, 2008; Shea et al., 2011; English et al., 2015). As the maternal phenotype is itself an evolving variable and a function of a mother's genes, her environment and, possibly, the phenotype of previous ancestors, predicting when offspring are selected to rely on the maternal phenotype is more complicated. Moreover, information present in a maternal phenotype is necessarily affected by a time‐lag, as the environment experienced by offspring may well have changed relative to the environment experienced by the mother.

So when is a maternal phenotype informative about the offspring's environment? We predict that this is the case when two conditions are met: (1) the maternal phenotype becomes correlated with her own (maternal) environment and (2) in turn, the maternal environment is correlated with the environment experienced by her offspring. Although condition (2) depends on properties of the external environment (i.e., presence of an environmental autocorrelation; Vasseur and Yodzis, 2004; Kuijper et al., 2014), the correlation required in (1) depends on the nature of adaptation. For example, if individuals with phenotypes that more closely match their environment are also more likely to survive and reproduce, classical theory predicts that a correlation between the maternal phenotype and her environment readily arises (Price, 1970; McNamara and Dall, 2011). In addition, future mothers who are maladapted at birth may use adaptive within‐generational plasticity to produce an adult phenotype that matches prevailing conditions more closely, again leading to a correlation between the maternal phenotype and her environment. Consequently, we predict that both natural selection and adaptive plasticity are likely to positively affect the evolution of maternal effects, but a model is necessary to quantify their relative importance.

The current study builds on a set of previous quantitative genetics models (Hoyle and Ezard, 2012; Ezard et al., 2014; Prizak et al., 2014) to assess how within‐generational plasticity and maternal effects affect adaptation. Although previous predictions were based on the differential fitness of an evolutionarily *constant* maternal effect, here we derive evolutionary dynamics that track the evolution of maternal effects from scratch. Consequently, the current study is the first to compare the evolution of (1) maternal effects, (2) direct genetic effects, and (3) within‐generational plasticity within a single framework. Results are corroborated using a recently published individual‐based simulation model of evolving maternal effects (Kuijper et al., 2014), which allows us to extend our model to a broader range of biologically relevant conditions, ‐such as strong selection,which are difficult to model analytically.

We model the evolution of within‐generational plasticity and maternal effects across a number of environments: first we focus on a baseline scenario in which maternal effects evolve in a constant environment. Next, we assess whether maternal effects facilitate adaptation to novel environments, by considering an environment that changes toward a novel optimum (Lande, 2009; Hoyle and Ezard, 2012). Finally, we study a temporally fluctuating environment that changes periodically according to a sinusoidal cycle (Ezard et al., 2014). Periodic environments could, for example, reflect regular cycles of host–parasite coadaptation or seasonal environments. In addition, a periodic environment also provides a straightforward, deterministic means to vary the degree of environmental autocorrelation between subsequent generations, which we predict to be key to the evolution of maternal effects. In the discussion, we show, however, that conclusions from the periodic environment also extend to other environments such as temporally varying stochastic environments (see also Kuijper et al., 2014) and spatial environments.

## The model

The current analysis is based on a previous quantitative genetics model by Lande and coworkers (Lande, 2009; Chevin et al., 2010) who studied the evolution of phenotypic plasticity by means of a linear reaction norm with elevation $a_{t}$ (reflecting the impact of an individual's genotype on its phenotype when plasticity and maternal effects are absent) and slope $b_{t}$. To this model, we add the evolution of a “trait based” maternal effect coefficient $m_{t}$ (McGlothlin and Brodie, 2009; McGlothlin and Galloway, 2013), which has been the subject of several previous quantitative genetics models of maternal effects (Kirkpatrick and Lande, 1989; Lande and Kirkpatrick, 1990; Hoyle and Ezard, 2012; Ezard et al., 2014). Although these previous studies assumed that $m_{t}$ is a constant parameter, here we allow $m_{t}$ itself to evolve (as well as $a_{t}$ and $b_{t}$).

### PHENOTYPES

An individual's phenotype $z_{t}$ at time *t* is given by

(1) $$z_{t}=a_{t}+b_{t}{ɛ}_{t-\tau }+m_{t}z_{t-1}^{*}+e_{t},$$

where $a_{t}$ is the elevation of the genotypic reaction norm in the reference environment ${ɛ}_{t-\tau }=0$, $b_{t}$ is the genetically encoded slope of the reaction norm that determines the plastic phenotypic response to the environment ${ɛ}_{t-\tau }$, where τ indicates the time point prior to selection at which an individual is exposed to environmental information (Lande, 2009), and $m_{t}$ is a maternal effect coefficient that reflects a linear, transgenerational reaction norm (Smiseth et al., 2008; Uller, 2012) on the parental phenotype $z_{t-1}^{*}$. Here, the * denotes a phenotypic value after survival selection, which is assumed to take place prior to reproduction. Our model assumes that maternal effects $m_{t}$ are controlled by the offspring, which describes a scenario in which offspring evolve their sensitivity to parental signals comprised in the parental phenotype (Müller et al., 2007; Smiseth et al., 2008). For example, the phenotype *z* could reflect a hormone titer (Groothuis and Schwabl, 2008; Gil, 2008), where offspring hormone titers $z_{t}$ are, partially, determined by the parental hormone titer $z_{t-1}^{*}$. $m_{t}$ reflects then the strength of the transgenerational norm of reaction (Uller, 2008; Smiseth et al., 2008) with which the offspring hormone titer depends on the parental hormone titer. Putatively, $m_{t}$ could reflect therefore the density of maternal hormone binding sites in the offspring's tissue that produces the hormone in question (e.g., endocrine glands).

Additionally, equation (1) shows that our model differs from some models of indirect genetic effects (e.g., Cheverud, 1984; Wolf and Brodie, 1998; Wolf et al., 1998), which assume the presence of maternal genetic effects (Rossiter, 1996), where the mother's genotype is the transgenerational aspect that affects the offspring's phenotype. However, the product $m_{t}z_{t-1}^{*}$ in equation (1) shows that it is the maternal phenotype (not genotype) that affects the offspring's phenotype, leading to “cascading” maternal effects (McGlothlin and Galloway, 2013) as the maternal phenotype itself is a function of the phenotypes of previous ancestors.

### FITNESS

Following standard quantitative genetics analyses (e.g., Lande 1976, 2009; Chevin et al. 2010), we assume a Gaussian fitness function, in which the fitness *W* of an individual in generation *t* decreases nonlinearly with the distance that its phenotype $z_{t}$ is displaced from the phenotypic optimum $\theta_{t}$. To assess the role of constraints, we also assume that both phenotypic plasticity $b_{t}$ (DeWitt et al., 1998; Chevin et al., 2010; Auld et al., 2010) and maternal effects $m_{t}$ impose survival costs on their bearers, which increase nonlinearly away from $(b_{t},m_{t})=0$. Costs of expressing the maternal effect are incurred by the offspring, as they control the expression of $m_{t}$ (see section “Phenotypes” above).

Consequently, individual fitness in generation *t* is given by

(2) Wzt,bt,mt=W max  exp −zt−θt22ωz2−bt22ωb2−mt22ωm2,

where $\omega_{z}$ is a parameter that is inversely proportional to the strength of selection that acts on phenotypes $z_{t}$ away from the selective optimum $\theta_{t}$. Similarly, $\omega_{b}$ is an inverse measure of the cost of phenotypic plasticity $b_{t}$ and $\omega_{m}$ is an inverse measure of the cost of maternal effects $m_{t}$. *W*_max_ is the maximum fitness of an individual, which we set to 1 throughout (without loss of generality). From the expression of $W(z_{t},b_{t},m_{t})$ we can then approximate mean fitness ${\bar{W}}_{t}$ (see Appendix) for weak selection on *z*, *b*, and *m* as

(3) W¯t=W max γzγbγmωz2ωb2ωm2× exp −12γzz¯t−θt2+γbb¯t2+γmm¯t2+O1ω4,

where $\gamma_{z}=1/\left(\omega_{z}^{2}+\sigma_{z_{t}}^{2}\right)$, $\gamma_{b}=1/\left(\omega_{b}^{2}+G_{bb}\right)$, $\gamma_{m}=1/\left(\omega_{m}^{2}+G_{mm}\right)$, $\sigma_{z_{t}}^{2}$ is the phenotypic variance at time *t* and $G_{bb}$ and $G_{mm}$ are the additive genetic variances in phenotypic plasticity and maternal effect coefficient, respectively. $O(1/\omega^{4})$ reflects the contribution to mean fitness of any higher order terms of the inverse selection strength parameter $\omega_{z}^{2}$ and inverse cost measures $\omega_{b}^{2}$ and $\omega_{m}^{2}$. As we assume selection to be weak ($\omega_{z}^{2}$ large) and costs to be small ($\omega_{b}^{2}$ and $\omega_{m}^{2}$ large), the contribution of these higher order terms is considered to be negligibly small in the analysis below.

### ENVIRONMENTAL CHANGE

We assume that the optimum phenotype $\theta_{t}$ is given by a linear function of the environment ${ɛ}_{t}$ at time *t*:

(4) $$\theta_{t}=A+B{ɛ}_{t},$$

where A=0 is the baseline level of the phenotypic optimum, and *B* is a parameter that reflects how changes in the environment affect the phenotypic optimum.

We study two different scenarios of environmental change. In the first scenario, we study the importance of maternal effects in the case in which a population experiences a single sudden, shift to a novel environment (as in Lande 2007; Hoyle and Ezard 2012). ${ɛ}_{t}$ is given by

(5) $${ɛ}_{t}=U_{t}\delta +\xi_{t},$$

where $U_{t}$ is a unit step function (which shifts from 0 to 1 at t=t switch ) that governs the sudden environmental change by an amount δ, and $\xi_{t}$ represents background environmental stochasticity, given by an autocorrelated Gaussian time series with autocorrelation ρ. In the second scenario, we study a periodically fluctuating environment in which environmental change is given by a discrete‐time sinusoid

(6) ɛt= sin ft+ξt,

where *f* is the rate of environmental change.

### EVOLUTIONARY DYNAMICS

The evolutionary dynamics are then described according to the multivariate breeder's equation (Lande, 1979), where we assume that pleiotropic mutations and linkage disequilibria are absent and selection is weak, so that genetic correlations between $a_{t}$, $b_{t}$, and $m_{t}$ can be ignored relative to the size of the respective additive genetic variances $G_{aa}$, $G_{bb}$, and $G_{mm}$. We then have

(7) Δa¯tb¯tm¯t=Gaa000Gbb000Gmm∂∂a¯t∂∂b¯t∂∂m¯t ln W¯t.

Substituting for  ln W¯t from equation (3) then yields

(8a) $$\Delta {\bar{a}}_{t}=\frac{G_{aa}}{\omega_{z}^{2}}\left[-\left({\bar{z}}_{t}-\theta_{t}\right)\frac{\partial {\bar{z}}_{t}}{\partial {\bar{a}}_{t}}-\frac{1}{2}\frac{\partial \sigma_{z_{t}}^{2}}{\partial {\bar{a}}_{t}}\right]+O\left(\frac{1}{\omega^{4}}\right)$$

(8b) $$\begin{array}{c} \Delta {\bar{b}}_{t}=\frac{G_{bb}}{\omega_{z}^{2}}\left[-\left({\bar{z}}_{t}-\theta_{t}\right)\frac{\partial {\bar{z}}_{t}}{\partial {\bar{b}}_{t}}-\frac{1}{2}\frac{\partial \sigma_{z_{t}}^{2}}{\partial {\bar{b}}_{t}}-\frac{\omega_{z}^{2}{\bar{b}}_{t}}{\omega_{b}^{2}}\right]+O\left(\frac{1}{\omega^{4}}\right) \end{array}$$

(8c) $$\begin{array}{c} \Delta {\bar{m}}_{t}=\frac{G_{mm}}{\omega_{z}^{2}}\left[-\left({\bar{z}}_{t}-\theta_{t}\right)\frac{\partial {\bar{z}}_{t}}{\partial {\bar{m}}_{t}}-\frac{1}{2}\frac{\partial \sigma_{z_{t}}^{2}}{\partial {\bar{m}}_{t}}-\frac{\omega_{z}^{2}{\bar{m}}_{t}}{\omega_{m}^{2}}\right]+O\left(\frac{1}{\omega^{4}}\right). \end{array}$$

In the Appendix, we calculate the derivatives $\partial {\bar{z}}_{t}/\partial {\bar{x}}_{t}$ and $\partial \sigma_{z_{t}}^{2}/\partial {\bar{x}}_{t}$ for all the three traits ${\bar{x}}_{t}\in \left\{{\bar{a}}_{t},{\bar{b}}_{t},{\bar{m}}_{t}\right\}$, which requires explicit expressions for ${\bar{z}}_{t}$ and $\sigma_{z_{t}}^{2}$ that we derive in equations (A5, A11).

As maternal effects cause phenotypes to depend recursively on their mother's phenotype (and thus on the phenotypes of all previous ancestors, e.g., Kirkpatrick and Lande, 1989; McGlothlin and Galloway, 2013), finding any analytical solutions to equation (7) becomes prohibitively difficult. Here, we therefore iterate the system in (7) numerically.

For each run, the initial values for ${\bar{a}}_{t=0},{\bar{b}}_{t=0},{\bar{m}}_{t=0}$ are set at $1\times 10^{-4}$. To assess whether our conclusions presented below are sensitive to initial conditions, we also ran iterations for all possible combinations of the following sets of starting values: ${\bar{a}}_{t=0}=\left\{-2,-1,1\times 10^{-4},1,2\right\},\;{\bar{b}}_{t=0}=\left\{-2,-1,1\times 10^{-4},1,2\right\},$ and ${\bar{m}}_{t=0}=\left\{-0.9,-0.5,1\times 10^{-4},0.5,0.9\right\}$. Note that we did not consider values of $|{\bar{m}}_{t=0}|\geq 1.0$, as phenotypic variances tend to go to infinity for these values (equation [A28] in Kirkpatrick and Lande, 1989). All numerical solutions converged to the evolutionary trajectories presented below.

### INDIVIDUAL‐BASED SIMULATIONS

To assess the robustness of our analytical results, we compared them to results derived from individual‐based simulations. We simulate a sexually reproducing population of N=5000 hermaphrodites with discrete generations. Each individual bears three unlinked, diploid loci that code for loci $a_{t}$, $m_{t}$, and $b_{t}$, respectively. The life cycle includes three stages: birth, survival, and reproduction. Upon birth, individuals develop their phenotype $z_{t}$ according to equation (1), potentially based on the phenotype of their mother (in case $m_{t}\neq 0$). Subsequently, individuals survive with probability w≡w min +(1−w min )W(zt,mt,bt) with $W(z_{t},m_{t},b_{t})$ given in equation (2). Here, the constant w min =0.1 serves to prevent premature extinction of the population away from the phenotypic optimum. Consequently, surviving individuals reproduce by randomly choosing another surviving individual as a sperm donor and go on to produce a clutch of N/n surv  offspring, to maintain a constant population size. Upon fertilization, each of the two alleles coding for traits $x_{t}\in \left\{a_{t},b_{t},m_{t}\right\}$ mutates with corresponding probabilities $\mu_{x}$. In case of a mutation, a value drawn from a normal distribution $N(0,\sigma_{x}^{2})$ is added to the old allelic value, resembling a continuum‐of‐alleles model (e.g., Kimura and Crow, 1964; Kimura, 1965). The two alleles that underlie each locus interact additively. Simulations were run for 50, 000 generations. Simulations are coded in C and can be downloaded from http://dx.doi.org/10.5281/zenodo.16685.

## Results

### RESULT 1: ONLY NEGATIVE MATERNAL EFFECTS EVOLVE IN CONSTANT ENVIRONMENTS

First, we consider a baseline case in which within‐generational plasticity $b_{t}$ and maternal effects $m_{t}$ are both absent, so that adaptation occurs through evolution of $a_{t}$ only. In addition, the selective optimum is constant over time, i.e., $\theta \equiv \theta_{t}$, which unsurprisingly favors the mean genetic effect to coincide with the optimum $\hat{\bar{z}}=\hat{\bar{a}}=\theta$. We then consider whether maternal effects are able to evolve by allowing for a slight amount of genetic variation in maternal effects $1>G_{mm}>0$. When ${\bar{z}}_{t-1}^{*}\approx {\bar{z}}_{t}$ as expected in a constant environment, we can then approximate the initial evolutionary change of a novel maternal effect (in the absence of plasticity) as

(9) $$\Delta \bar{m}{|}_{\bar{m}=0,\bar{z}=\theta }\;=-\frac{G_{mm}\left[4G_{aa}+{\bar{z}}_{t}^{2}G_{mm}\left(12+G_{mm}\right)\right]}{8\omega_{z}^{2}\left(1-G_{mm}\right)}.$$

As all coefficients within brackets are positive, this suggests that maternal effects always evolve toward negative values in stationary environments. Indeed, this confirms previous results (Hoyle and Ezard, 2012) that stationary populations selectively favor negative maternal effects as a means to reduce the amount of phenotypic variance (e.g., see Figure 3.1 in Hoyle and Ezard, 2012).

In the current situation, where maternal effects are allowed to evolve, we show in the Appendix that equilibrium solutions in our model must always correspond to a negative mean maternal effect, $\bar{m}<0$. For small values of $G_{mm}$ in the absence of costs of plasticity and maternal effects, this can again be interpreted as minimizing the phenotypic variance, since then $\bar{z}\approx \theta$ from equation (A24) and from the expression of $\gamma_{z}$ in the equation for mean fitness (3) the “variance load” is the factor that reduces population mean fitness in this case. It can be shown (eq. A28) that at equilibrium in constant environments, ${ɛ}_{t}\equiv ɛ$, the phenotypic variance is approximately

(10) $$\begin{array}{ccc} \sigma_{z_{t}}^{2}\approx \frac{1}{1-G_{mm}-{\bar{m}}^{2}} & & \left[\frac{2+\bar{m}}{2-\bar{m}}\left(G_{aa}+G_{bb}{ɛ}^{2}+G_{mm}{\bar{z}}^{2}\right)\right.\\ & & +\left.\frac{{\bar{z}}^{2}G_{mm}^{2}}{{\left(2-\bar{m}\right)}^{2}}+\sigma_{e}^{2}\right]. \end{array}$$

**Figure 1:**
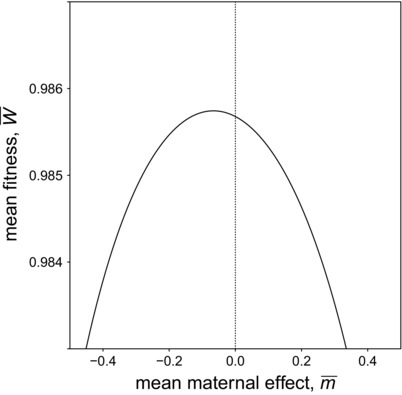
Variation of population mean fitness with mean maternal effect in a constant environment, when the mean phenotype is optimal and in the absence of costs of plasticity or maternal effects. For the parameter values used subsequently in Figure 2, it can be seen that mean fitness is maximized at negative $\bar{m}$. Parameters: $G_{aa}=0.1,\;G_{bb}=0.045,\;G_{mm}=0.005,\;\omega_{z}^{2}=40,\;A=0,\;B=2,\;\theta =10,\;\sigma_{e}^{2}=1,\;\omega_{m}^{2}=\omega_{b}^{2}=100$.

We show in Figure 1 how the fitness varies with the mean maternal effect for a case in which $G_{mm}$ is small and costs of maternal effects are absent: it can be seen that the maximum fitness is found for negative $\bar{m}$. For fixed maternal effects, Hoyle and Ezard (2012) showed that the minimum variance load always occurs for negative *m*.

When there is a cost of maternal effects, minimizing it is traded off against minimizing the phenotypic variance (eq. 8c). When $G_{mm}$ is not so small that we can approximate $\bar{z}\approx \theta$, equation (8c) also shows that there are trade‐offs between minimizing the phenotypic variance, minimizing the cost of maternal effects, and reaching the optimal phenotype (see Fig. S2).

### RESULT 2: MATERNAL EFFECTS EVOLVE TO TRANSIENTLY POSITIVE VALUES FOLLOWING EXTREME ENVIRONMENTAL SHIFTS

**Figure 2:**
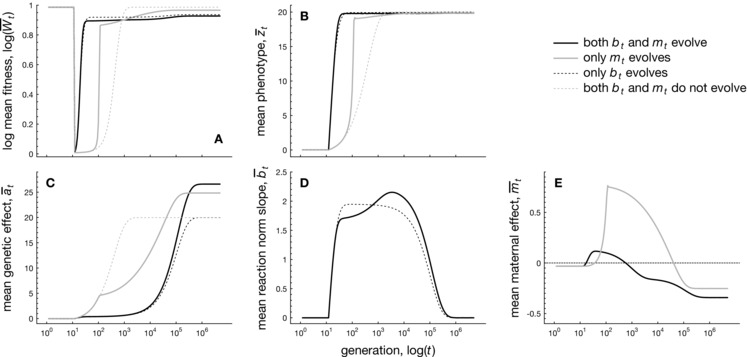
Numerical iterations showing adaptation to a sudden shift in the environment ${ɛ}_{t}$ at t=10 for different populations that vary in the presence or absence of within‐generational plasticity or maternal effects, while the elevation $a_{t}$ is always allowed to evolve. Solid black lines: both within‐generational plasticity and maternal effects $b_{t}$ and $m_{t}$ are allowed to evolve. Solid gray line: only maternal effects $m_{t}$ are allowed to evolve (no plasticity). Dashed black line: only plasticity $b_{t}$ is allowed to evolve (no maternal effects). Dashed gray lines: neither $b_{t}$ and $m_{t}$ are allowed to evolve (i.e., only the elevation $a_{t}$ evolves). Panel A: change in population mean fitness $W_{t}$. Panel *B*: evolution of the mean phenotype ${\bar{z}}_{t}$. Panel C: the mean elevation ${\bar{a}}_{t}$. Panel D: the mean level of within‐generational plasticity ${\bar{b}}_{t}$ (reaction norm slope). Panel E: the mean maternal effect coefficient ${\bar{m}}_{t}$ . Parameters: $G_{aa}=0.1,\;G_{bb}=0.045,\;G_{mm}=0.005,\;\omega_{z}^{2}=40,\;A=0,\;B=2,\;\sigma_{\xi }^{2}=0.01,\;\rho =0.5,\;\delta =10,\;\tau =0.25,\;\sigma_{e}^{2}=1,\;\omega_{m}^{2}=\omega_{b}^{2}=100$.

Next, we consider an environment that changes according to a rapid shift, remaining constant thereafter (see also Lande 2009; Hoyle and Ezard 2012). Figure 2 shows the course of evolution during a rapid environmental shift (taking place during a single generation) for different populations that vary in the presence of plasticity $b_{t}$ and maternal effects $m_{t}$. Paleoclimatic data have shown, for example, that such abrupt environmental shifts—taking less than 3 years—have occurred during Late Pleistocene (Steffensen et al., 2008; Hof et al., 2011).

#### Speed of adaptation to an extreme shift

Populations in which both evolving plasticity and maternal effects are present show the quickest recovery in terms of mean fitness $\bar{W}$ (solid black line in Fig. 2A). Populations in which only maternal effects are present recover more slowly (solid gray line), also relative to populations in which only phenotypic plasticity is present (dashed black line), but still recover ten‐folds of generations faster relative to populations that only have genetic effects (dashed gray line). Consequently, Figure 2 corroborates previous findings that maternal effects are advantageous in changing environments (Räsänen and Kruuk, 2007; Uller, 2008; Hoyle and Ezard, 2012), with combinations of maternal effects and phenotypic plasticity providing the fastest adaptation to change (Hoyle and Ezard, 2012; Ezard et al., 2014). Individual‐based simulations result in very similar evolutionary trajectories to those shown in Figure 2 (see Fig. S1).

#### The evolution of maternal effects during extreme shifts

During the abrupt environmental shift, $\bar{m}$ rapidly evolves to positive values, after which it remains positive for several hundred generations before settling again at negative values (Fig. 2E). Such transiently positive values of $\bar{m}$ occur regardless of the sign and magnitude of the environmental shift δ and are robust to strong costs $\omega_{m}^{-2}$ (see Fig. S3). To understand this transient evolutionary pattern of $\bar{m}$, note from eq. (1) that maternal effects result in a contribution $m_{t}z_{t-1}^{*}$ from a surviving mother's phenotype $z_{t-1}^{*}$ to the offspring's phenotype $z_{t}$. As a surviving mother is likely to have a phenotype *z* that lies closer to the novel optimum (compared to phenotypes of non‐survivors), offspring are selectively favored to copy the beneficial maternal phenotype by evolving a positive maternal effect. Note, however, that $\bar{m}$ is much smaller (yet still positive) in the presence of phenotypic plasticity $\bar{b}$ (black line in Figs. 2E and S3D–F), as the presence of plasticity reduces the necessity of relying on maternal effects for adaptation. Notwithstanding these lower levels of $\bar{m}$ in the presence of phenotypic plasticity, positive maternal effects are transiently advantageous for populations experiencing sudden environmental shifts.

Note that $\bar{m}$ also affects the magnitude of the elevation $\bar{a}$: populations with maternal effects show considerably higher values of $\bar{a}$ at the novel optimum relative to populations in which maternal effects are absent (Fig. 2C). Higher values of $\bar{a}$ occur because negative maternal effects at equilibrium not only reduce the phenotypic variance, but also reduce the offspring's phenotype by a factor $m_{t}z_{t-1}^{*}$. Although such a reduction is less of an issue in the original environment in which $z_{t-1}^{*}$ is close to zero, such reductions matter in the novel environment and are compensated through the evolution of a higher level of $a_{t}$ relative to populations in which maternal effects are absent.

#### Gradually changing environments

When environmental shifts occur at slower timescales of 100 or 1000 years (as is the case for current global warming; e.g., PAGES 2k Consortium, 2013), we find a similar pattern to that in Figure 2 (see Fig. S4). Only when environmental change occurs at a much slower timescale (10,000 years and beyond), do we find that maternal effects and phenotypic plasticity attain transient values of a much more modest magnitude (Fig. S4). In the latter case, changes in the underlying elevation $a_{t}$ are sufficient to account for most of the change, avoiding the slight costs associated with maternal effects or phenotypic plasticity. Consequently, maternal effects and phenotypic plasticity evolve more readily with more rapid environmental shifts.

### RESULT 3: STRONG SELECTION AND LIMITED PLASTICITY FAVOR MATERNAL EFFECTS IN FLUCTUATING ENVIRONMENTS

#### Weak selection

**Figure 3:**
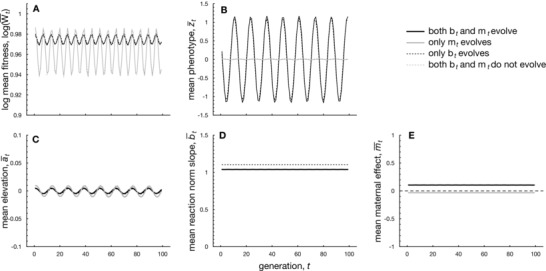
Numerical iterations showing adaptation to sinusoidally changing environment with frequency f=0.5. Panels as in Figure 2. Parameters: $G_{aa}=0.1,\;G_{bb}=G_{mm}=0.045,\;\omega_{z}^{2}=40,\;A=0,\;B=2,\;\sigma_{\xi }^{2}=0.01,\;\rho =0.5,\;\tau =0.25,\;\sigma_{e}^{2}=1,\;\omega_{m}^{2}=\omega_{b}^{2}=100$. The amplitude of the sine wave is 1.

**Figure 4:**
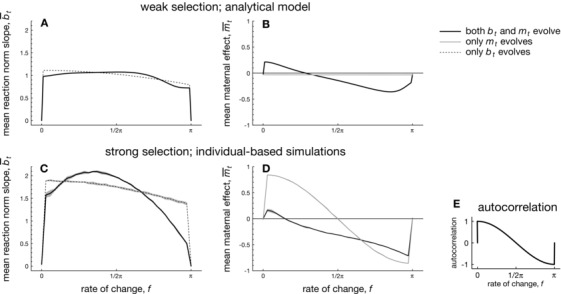
The evolution of mean within‐generational plasticity $\bar{b}$ and mean maternal effects $\bar{m}$ while varying the frequency of environmental change *f*. Panels A and B: evolution of $\bar{a}$, $\bar{m,}$ and $\bar{b}$ according to the analytical model when selection on the overall phenotype is weak (i.e., $\omega_{z}^{2}=40$). Panels C and D: evolution of $\bar{a}$, $\bar{m,}$ and $\bar{b}$ according to the analytical model when selection on the overall phenotype is strong $\left(\omega_{z}^{2}=0.7\right)$, with shading representing the standard deviation over 10 replicate simulation runs for each value of *f*. Panel E: the autocorrelation in selective conditions between the maternal and offspring generations, which is approximately $\mathrm{cor}\left(\theta_{t},\theta_{t+1}\right)\approx \mathrm{cor}(\sin\left(ft\right),\sin\left(f\left(t+1\right)\right)$ when the variance $\sigma_{\xi }^{2}$ of the background environmental stochasticity is small, as is assumed here. Parameters: $A=0,\;B=2,\sigma_{\xi }^{2}=0.01,\;\rho =0.5,\;\tau =0.25,\;\omega_{m}^{2}=\omega_{b}^{2}=100$. Parameters for the analytical model: $G_{aa}=0.1,\;G_{bb}=G_{mm}=0.045$. Parameters for individual‐based simulations: $\mu_{a}=\mu_{b}=\mu_{m}=0.02,\;\sigma_{\mu_{b}}^{2}=\sigma_{\mu_{m}}^{2}=\sigma_{\mu_{z}}^{2}=0.0025,\;\sigma_{e}^{2}=1$.

Next, we focus on populations that endure a continuously fluctuating environment given by a sinusoidal function with frequency *f*. When selection is weak and change is relatively slow (f=0.5), Figure 3B shows that populations with within‐generational plasticity (black lines) are more successful at adapting to fluctuating environments than those without plasticity (gray lines). By contrast, maternal effects are less advantageous: in the absence of plasticity, $\bar{m}$ always evolves to negative values of a very small magnitude (Figs. 3E and 4). When both plasticity and maternal effects are present, Figure 3 shows that $\bar{m}$ becomes weakly positive in slowly changing environments, in broad agreement with a previous investigation of evolutionarily fixed maternal effects in sinusoidal environments (Hoyle and Ezard, 2012; Ezard et al., 2014). Hence, positive maternal effect coefficients can be selected for in slowly changing, predictable environments. In general, however, the magnitude of $\bar{m}$ is small, showing that the maternal phenotype enhances adaptation only slightly when selection is weak (see Fig. 3A).

#### Weak selection and different rates of environmental change

Figure 4 depicts the evolved values of mean plasticity $\bar{b}$ and mean maternal effects $\bar{m}$, while varying the rate *f* of environmental change when phenotypic selection is weak. Note that varying *f* from 0 to π causes the autocorrelation in selective conditions experienced by mothers and offspring to vary from positive to negative (see Fig. 4E), while the autocorrelation is approximately zero at $f\in \{0,\frac{1}{2}\pi ,\pi \}$ (at least when the amount of background environmental noise is small, as is assumed here).

For all frequencies *f*, the mean value of plasticity $\bar{b}$ evolves toward positive values of a considerable magnitude (regardless of whether plasticity coevolves with maternal effects or not), showing that environmental input to the phenotype is always selectively favored (Fig. 4A). By contrast, the mean maternal effect $\bar{m}$ is restricted to much smaller values: when maternal effects evolve in the absence of phenotypic plasticity, $\bar{m}$ evolves to slight negative values for all frequencies *f* (gray line in Fig. 4B). Maternal effects evolve to near‐zero values because selection is weak: consequently, the distribution of maternal phenotypes $p(z_{t-1}^{*})$ is broadly scattered around the selective optimum $\theta_{t-1}$, so that the maternal phenotype provides little information about the location of the selective optimum to offspring. As in the constant environment, $\bar{m}$ therefore merely evolves to slight negative values that reduce phenotypic variance.

By contrast, when maternal effects coevolve with phenotypic plasticity (black line in Fig. 4B), $\bar{m}$ evolves to slightly larger values: it attains positive values when environmental fluctuations are weak (i.e., when maternal and offspring environments are strongly positively correlated) and attains negative values in more rapidly fluctuating environments (i.e., when maternal and offspring environments are poorly or negatively correlated). The presence of within‐generational plasticity is conducive to the evolution of maternal effects, as plasticity brings the maternal phenotype closer toward the phenotypic optimum $\theta_{t-1}$. As a result, the distribution of maternal phenotypes $p(z_{t-1}^{*})$ is now more informative to offspring about the location of the selective optimum, relative to populations in which plasticity is absent.

However, the presence of within‐generational plasticity raises the question of why maternal effects evolve at all, as plasticity itself may provide a sufficient means to achieve adaptation. This would indeed have been the case, were it not that slight constraints act on plasticity (Fig. 4 assumes a small cost $\omega_{b}^{2}=100$ and a slight time lag τ=0.25), thereby selectively favoring maternal effects. If plasticity is unconstrained, however, it can be shown that maternal effects always evolve to slight negative values for all frequencies *f*, reflecting that maternal effects are not involved in adaptation to fluctuating environments. Consequently, the presence of within‐generational plasticity is conducive to the evolution of maternal effects when selection is weak, provided that plasticity itself is constrained.

#### Strong selection

Figure 4C shows that values of phenotypic plasticity $\bar{b}$ are much larger when selection is strong (here $\omega_{z}^{2}=0.7$), as individuals are under stronger selection to use environmental information to match the fluctuating environment. Regarding maternal effects, we find that when *m* evolves together with plasticity, a qualitatively similar pattern occurs as for the case of weak selection (compare Fig. 4B and D): maternal effects evolve to slight positive values in environments characterized by strong, positive autocorrelations between subsequent generations (Fig. 4E), whereas they evolve to negative values otherwise. Moreover, negative values of $\bar{m}$ can be substantial in case the environment is sufficiently negatively correlated close to f=π.

When maternal effects evolve in the absence of phenotypic plasticity, we find that strong selection favors maternal effects of a substantial magnitude (gray line in Fig. 4D). Interestingly, maternal effects evolve to be large and positive in slowly changing environments, which are characterized by a positive environmental autocorrelation between subsequent generations (Fig. 4 E). By contrast, in rapidly changing environments maternal effects evolve to negative values of a substantial magnitude, again in line with the environmental autocorrelation. To conclude, the strength of phenotypic selection matters considerably to the evolution of maternal effects, as only slight negative maternal effects were found in a corresponding scenario of weak selection (compare gray lines in Fig. 4B and D). Strong selection is conducive to the evolution of maternal effects, as it gives rise to a distribution of maternal phenotypes $p(z_{t-1}^{*})$ that is closely centered around the selective optimum $\theta_{t-1}$. As a result, the maternal phenotype is more informative about the location of the selective optimum to offspring.

#### Varying both the strength of selection and costs of plasticity

**Figure 5:**
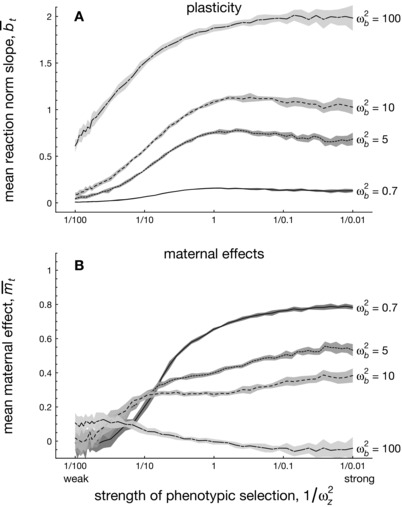
Individual‐based simulations showing the differential sensitivity of plasticity (panel A) and maternal effects (panel B) to the strength of phenotypic selection $\omega_{z}^{-2}$ in a slowly fluctuating environment (f=0.5). Each lines reflect different costs of plasticity $\omega_{b}^{-2}$. Phenotypic plasticity $\bar{b}$ readily evolves to appreciable values even when selection on the overall phenotype is very weak, unless the evolution of $\bar{b}$ is checked by considerable costs of plasticity (low values of $\omega_{b}^{2}$, bottom lines in panel A). By contrast, panel B shows that maternal effects $\bar{m}$ only evolve to significant values when selection on the overall phenotype is very strong, even when plasticity is constrained by strong costs $\omega_{b}^{-2}$. Parameters: $A=0,\;B=2,\sigma_{\xi }^{2}=0.01,\;\rho =0.5,\;\tau =0.25,\;\omega_{m}^{2}=100,\;\mu_{a}=\mu_{b}=\mu_{m}=0.02,\;\sigma_{\mu_{b}}^{2}=\sigma_{\mu_{m}}^{2}=\sigma_{\mu_{z}}^{2}=0.0025,\;\sigma_{e}^{2}=1$. Shaded range reflects standard deviations over 10 replicate simulation runs for each of 35 different values of $\omega_{z}^{2}$ .

Both the strength of phenotypic selection and the presence of plasticity appear to affect the evolution of maternal effects. Figure 5 generalizes these findings, by varying the strength of phenotypic selection (measured by $\omega_{z}^{-2}$) and the magnitude of plasticity (by varying costs of plasticity, $\omega_{b}^{-2}$). For a slowly fluctuating environment (f=0.5), Figure 5A shows that when plasticity has small costs (i.e., $\omega_{b}^{2}=100$), mean plasticity $\bar{b}$ readily attains substantial values, even when selection on the overall phenotype is still very weak. By contrast, the same does not occur for maternal effects (Fig. 5B): when a maternal effect imposes only slight costs (Fig. 5 assumes $\omega_{m}^{2}=100$ throughout), the evolved values of maternal effects are all small when selection is very weak to modestly strong (i.e., $1/100>\omega_{z}^{-2}>1/10$). Moreover, for this range of selection pressures $\omega_{z}^{2}$, we find that slight positive values of maternal effects occur for those populations in which plasticity only bears a slight cost (long‐dashed line in Fig. 5B), whereas small, negative maternal effects evolve when plasticity is extremely costly (solid line in Fig. 5B). Hence, this conforms to our previous finding that, in case of weak selection, the presence of plasticity is conducive to the evolution of maternal effects.

When selection on the overall phenotype becomes progressively stronger, however, Figure 5B shows that maternal effects $\bar{m}$ evolve to more substantial, positive values to match the slowly changing environment (f=0.5; see also Fig. 4). Such large values of $\bar{m}$ only occur, however, when phenotypic plasticity is sufficiently constrained by costs, whereas maternal effects evolve to negligible values otherwise. Again, when selection is strong, plasticity hampers rather than enhancing maternal effects. We can thus conclude two things from Figure 5: the first is that the phenotypic plasticity and maternal effects affect each other highly asymmetrically. Although the presence of phenotypic plasticity substantially affects the magnitude of maternal effects, maternal effects themselves have only a slight impact on phenotypic plasticity. Moreover, we find that for a similar level of cost, maternal effects require stronger phenotypic selection to evolve to significant values in comparison to phenotypic plasticity.

#### Developmental constraints

As noted previously, Figure 5 shows that constraints on plasticity—in the form of costs—can substantially affect the evolution of maternal effects. The last part of our results consider whether the same holds when plasticity is otherwise constrained, for example through constraints acting on an individual's perception of the environment. For example, some organisms' response to the environment may be subject to a time‐lag, 0<τ<1. This would reflect a scenario in which a phenotype is only plastic during early development (Lande, 2009; Hoyle and Ezard, 2012), while an individual is unable to adjust its phenotype to later environmental cues at the time when it endures selection (occurring a fraction τ of a generation after development).

**Figure 6:**
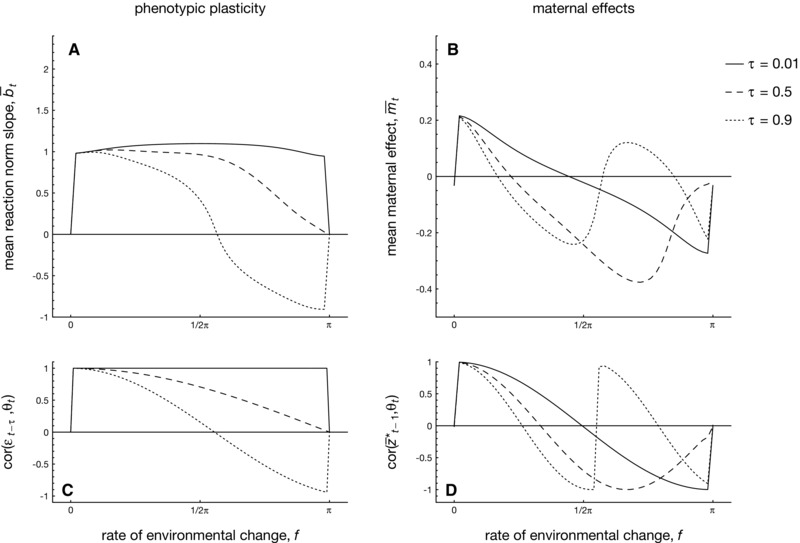
The simultaneous evolution of mean plasticity $\bar{b}$ (Panel A) and mean maternal effects $\bar{m}$ (Panel B) for three levels of the developmental time‐lag τ, when selection is weak ($\omega_{z}^{2}=40$). Panel C: correlation between an individual's developmental environment ${ɛ}_{\left\{t-\tau \right\}}$ and the selective optimum experienced by that same individual. Panel D: correlation between a maternal phenotype and the selective optimum experienced by her offspring. Parameters: $A=0,\;B=2,\sigma_{\xi }^{2}=0,\;\omega_{m}^{2}=\omega_{b}^{2}=100$, $G_{aa}=0.1,\;G_{bb}=G_{mm}=0.045$.

Figure 6A shows that a small developmental time lag τ=0.01 causes plasticity to achieve positive values for all frequencies *f* of environmental change, as the perceived environmental information always closely matches an individual's selective conditions. When the time‐lag τ increases (e.g., τ=0.5, long‐dashed line), however, plasticity gradually decreases to 0 with increasing rates of environmental change or even becomes negative when τ=0.9 (fine‐dashed line). These values of plasticity can be understood by considering the correlation $\mathrm{cor}({ɛ}_{t-\tau },\theta_{t})$ between the developmental environment ${ɛ}_{t-\tau }$ perceived by an individual at time t−τ and the selective optimum $\theta_{t}$ it will experience, which obviously is affected by the value of the time‐lag τ. Figure 6C shows that plasticity evolves roughly according to the value of this correlation.

When considering the evolution of maternal effects, Figure 6B shows that, when the time‐lag is small to modest, the mean maternal effect $\bar{m}$ varies from positive to negative with increasing rates of environmental change, similar to what was observed in Figure 4B and D (which assumed a modest time lag τ=0.25). When the developmental lag τ is large, however (e.g., τ=0.9), $\bar{m}$ varies in a more complicated fashion, from positive to negative and then again from positive to negative. How can we explain these patterns? To understand the evolution of $\bar{m}$, Figure 6D shows the correlation  cor z¯t−1*,θtbetween the mean maternal phenotype after selection ${\bar{z}}_{t-1}^{*}$ and the selective optimum $\theta_{t}$. This correlation illustrates how the maternal phenotype lines up with the selective conditions that are experienced by offspring, and shows that the sign and magnitude of this correlation vary according to the rate of environmental change *f* and the value of τ. We find that the sign of mean maternal effect $\bar{m}$ evolves roughly in line with this correlation, although the actual magnitude of $\bar{m}$ is smaller.

## Discussion

As opposed to numerous studies that have assessed the consequences of a fixed maternal effect on other characters (Kirkpatrick and Lande, 1989; Wolf et al., 1999; Räsänen and Kruuk, 2007; Hoyle and Ezard, 2012; Ezard et al., 2014), this study is one of the first to assess the evolutionary dynamics of maternal effects themselves. Interestingly, our model shows that maternal effects are indeed anything but a static parameter: rather, the evolved magnitude and sign of maternal effects are sensitive to specific ecological and organismal features, such as the nature of environmental change, the strength of selection and the presence of other mechanisms that facilitate adaptation (such as phenotypic plasticity).

Focusing on the evolution of maternal effects, we find that rapid environmental shifts lead to the transient evolution of positive maternal effects of a large magnitude, during which maternal effects remain positive for several thousand generations (see Fig. 2). As highlighted in the results, the reason for the presence of such positive maternal effects is that an individual that manages to survive and reproduce is likely to have a phenotype that lies closer to the novel environmental optimum. Consequently, offspring that aim to adjust themselves to the novel environment benefit by attaining a similar phenotype to their parents, which is achieved through positive parental effects. Hence, the evolution of maternal effects in response to environmental shifts confirms well‐established verbal theories (Uller, 2008, 2012), which state that maternal effects evolve when the parental phenotype provides information about the offspring's future environment. We find that such transiently positive parental effects occur even when phenotypic plasticity is also present (although the effects are less pronounced). That maternal effects still exhibit a marked evolutionary response in the presence of phenotypic plasticity is due to the sudden nature of the shift: after the environmental perturbation has occurred, drastically larger values of the elevation *a* and the reaction norm *b* become selectively favored. However, as the evolution of larger values of *a* and *b* does not occur instantaneously, the evolution of maternal effects provides a powerful additional means of rapid adaptation to sudden changes in environmental conditions, as it allows the maternal phenotype closer to the optimum to influence the offspring's phenotype.

Results are strikingly different, however, in the context of periodically changing environments, where an environment never reaches a new equilibrium, but changes continuously. When selection is weak, we find the scope for maternal effects of a substantial magnitude to be only modest in fluctuating environments (e.g., Fig. 4B). The limited prevalence of maternal effects when selection is weak and plasticity is absent is in line with the notion that maternal effects will only evolve when the parental phenotype $z_{t-1}^{*}$ is informative about future environmental conditions (see also Uller, 2008; Fischer et al., 2011; Kuijper and Johnstone, 2013; Kuijper et al., 2014). When selection acting on the maternal phenotype is weak (and phenotypic plasticity is absent), the maternal phenotype $z_{t-1}^{*}$ will not correlate strongly with the prevailing environmental conditions, as individuals with phenotypes $z_{t-1}$ that lie very far away from the parental selective optimum $\theta_{t-1}$ are still able to survive and produce offspring. As the parental phenotype $z_{t-1}^{*}$ is thus largely uninformative about the selective environment to offspring, maternal effects are hardly relevant when selection is weak and plasticity is absent. By contrast, when plasticity is present, individuals adjust their phenotype to the prevailing environmental conditions, so that their phenotype $z_{t-1}$ becomes more closely aligned to the selective optimum $\theta_{t-1}$. As the parental phenotype $z_{t-1}^{*}$ is now more informative to offspring (at least when $\theta_{t-1}$ and $\theta_{t}$ are correlated), maternal effects of a larger magnitude evolve (Fig. 4B). Moreover, $\bar{m}$ generally evolves in line with the environmental autocorrelation (Fig. 4E, see also Kuijper et al. 2014), although this pattern becomes more complicated for species with long development times (see Fig. 6). The notion that plasticity can enhance the evolution of maternal effects corroborates similar findings by previous studies, which showed that certain combinations of plasticity and fixed maternal effects improve mean fitness (Hoyle and Ezard, 2012; Ezard et al., 2014).

When selection on the overall phenotype is stronger, we find that maternal effects achieve the largest values when plasticity is absent or severely constrained (e.g., Fig. 4D). This is unsurprising, as strong selection causes only those mothers to survive whose phenotype $z_{t-1}^{*}$ is very closely aligned to the selective optimum $\theta_{t}$. Consequently, strong selection makes the maternal phenotype predictive about the offspring environment (at least when $\theta_{t}$ and $\theta_{t+1}$ are correlated). Moreover, in the absence of plasticity, individuals are forced to rely on maternal effects as it is the only means of adaptation to a fluctuating environment. When plasticity is present, however, lower values of maternal effects evolve, as relying on plasticity (which constitutes a more direct source of environmental information, as opposed to indirect information through the maternal phenotype) is the preferred means of adaptation. As the relevance of strong selection in long‐term adaptation is generally considered to be limited (Kingsolver et al., 2001), the relevance of scenarios where maternal effects evolve to very large values remains to be empirically demonstrated. Nonetheless, in certain cases selection has been demonstrated to be strong (e.g., King et al., 2011), particularly in the realm of antagonistic coevolution. Based on our study, we would expect that maternal effects would be most easily detected in these contexts (see also Mostowy et al., 2012).

A general result emerging from this study is that phenotypic plasticity has a much stronger influence on adaptation than maternal effects (e.g., Figs. 2A and 3A). In relation to that, we also find a much larger impact of evolving phenotypic plasticity on the magnitude of maternal effects, whereas the reverse impact of maternal effects on plasticity is much more limited (e.g., see Fig. 4). That phenotypic plasticity is a more efficient means of adaptation is unsurprising, as plasticity relies on direct environmental information, whereas maternal effects necessarily rely on the maternal phenotype as an indirect source of environmental information. As a result, maternal effects only evolve when the maternal phenotype is sufficiently correlated with the environment that will be encountered by offspring, which in turn occurs only when selection is strong and an environmental autocorrelation is present between subsequent generations. As such conditions do not apply to direct environmental cues, it is not surprising that the role of maternal effects is thus more restrictive than phenotypic plasticity.

Our prediction that maternal effects have a rather limited role when selection is weak may well correspond with a recent meta‐analysis (Uller et al., 2013), which shows that there is only limited evidence of maternal effects facilitating adaptation to environmental change. In addition, another meta‐analysis finds that selection coefficients are, in fact, remarkably consistent over time, demonstrating that currently little evidence exists for either large selective shifts of a substantial magnitude or continuously fluctuating selection (Siepielski et al., 2013). Consequently, these lines of evidence would suggest that maternal effect coefficients *m* should evolve to be small and negative in the majority of cases. Indeed, empirical studies show that negative maternal effect coefficients appear to be the norm: (reviewed in Räsänen and Kruuk, 2007), only two cases of positive maternal effects have been found: maternal effects of adult body size on hatchling body size in Darwin's finches and great tits have coefficients m≈0.6 and m≈0.3, respectively (Lande and Price, 1989). By contrast, all other studies that measured maternal effects have found to be negative and relatively small (e.g., Falconer, 1965; Janssen et al., 1988; Schluter and Gustafsson, 1993; McAdam and Boutin, 2004). In addition, a number of studies have measured a negative correlation between direct genetic effects and maternal genetic effects (e.g., Cheverud, 1984; Wilson et al., 2005; Wilson and Réale, 2006; Räsänen and Kruuk, 2007; Kent et al., 2009), which often indicates that the actual maternal effects coefficient *m* is also negative (Falconer, 1965).

Although weak selection (Kingsolver and Diamond, 2011; Kingsolver et al., 2012; Siepielski et al., 2013) may be a fruitful explanation for the prevalence of negative maternal effects for the purpose of variance minimization (Hoyle and Ezard, 2012), this is of course not the whole story. Existing data on fluctuating selection is confounded by sampling biases (e.g., exclusion of unsuccessful years or small populations from analyses of selection) and typically only provides a brief snapshot in time (Siepielski et al., 2013). Also, the notion that major climatic variables (e.g., rainfall, temperature) are characterized by substantial temporal variation (Vasseur and Yodzis, 2004) shows that the ecological context of fluctuating selection is far from understood. In addition, although maternal effects have, on average, only slight consequences for offspring phenotypes (Uller et al., 2013), a number of undeniable examples exist where maternal phenotypes have clear transgenerational influences on offspring phenotypes (Gustafsson et al., 2005; Galloway and Etterson, 2007). It is imperative to tie these studies (and future ones) to information about (1) the strength of selection on the overall phenotype, (2) the strength of selection on phenotypic plasticity, and (3) the nature of environmental variation (e.g., positive vs. negatively correlated environments). In terms of measurable parameters, our study shows that the strength of selection on phenotypes needs to be substantial to give rise to maternal effects of a significant magnituede (i.e., phenotypic selection gradients $|\beta_{z}|\propto \frac{1}{\omega_{z}^{2}}>\frac{1}{2}$, see Fig. 5) and phenotypic plasticity needs to be costly (e.g., $|\beta_{b}|\propto \frac{1}{\omega_{b}^{2}}>\frac{1}{10}$), or constrained in other ways (see Auld et al., 2010). Lastly, the sign and magnitude of maternal effects is highly contingent on the nature of environmental variation, with positively correlated, or slowly and predictably changing, environments selecting for positive maternal effects, while negatively correlated, or rapidly changing, environments selectively favor negative maternal effects (see also Ezard et al., 2014; Kuijper et al., 2014).

To assess thoroughly whether variation in maternal effects can be tied to different ecological contexts, studies that measure intraspecific variation in maternal effect coefficients would be desirable. Although a number of studies have considered intraspecific variation in maternal effects (e.g., Mousseau, 1991; Williams, 1994), these studies only investigated phenotypic variation in offspring characters, but did not assess the strength and sign of maternal effects. Particularly suitable target species to measure intraspecific variation in maternal effects are those for which substantial detail about the genetic architecture is available through multigenerational pedigrees in different populations, as is the case for great tits *Parus major* (Vedder et al., 2013; Korsten et al., 2013). Next to that, measurements of parent–offspring correlations in multiple contexts (Lande and Price, 1989) would provide insight into the extent of maternal effects, which may be particularly interesting to assess variation in maternal effects in human populations (Kent et al., 2009; Stearns et al., 2010). In addition, experimental evolution (Kawecki et al., 2012), for example on offspring size, would provide a more rigorous approach to assessing the evolutionary properties of maternal effects, particularly when the rate of environmental fluctuations varies across experimental subpopulations.

Previous studies within the same framework suggest that our conclusions generalize to other contexts, such as stochastically fluctuating environments (Kuijper et al., 2014; Ezard et al., 2014). Indeed, Figure S5 shows that maternal effects also evolve in stochastically fluctuating environments. Similarly to our results in a periodic environment in which developmental delays are small (see Fig. 4), maternal effects evolve to positive (or negative) values in positively (or negatively) autocorrelated environments. In addition, stochastic models also allow to assess how maternal effects evolve in response to increasingly unpredictable environments (in which the autocorrelation ρ decreases toward 0), congruent with recent climate change (Hansen et al., 2012). Figure S5 shows that maternal effects rapidly decay to slight negative values that merely reduce phenotypic variance, with little transgenerational importance. Consequently, increasing climatic unpredictability is likely to reduce the scope for maternal effects in the long term.

Possible extensions to our model include the incorporation of spatial environmental variation. Given our previous results in temporally fluctuating environments (e.g., Fig. 4), we would expect that correlations between parental and offspring environments are also key to the evolution of maternal effects in spatial environments. In a simple spatial model (consisting of two different environments and a probability *d* with which individuals migrate to a different environment), we indeed find that correlations are again important (see Fig. S6): when dispersal d<0.5, maternal effects evolve to slight negative values as the majority of offspring remain in the natal environment and thus experience no change. By contrast, when the dispersal probability is higher (d≥0.5), maternal effects now evolve to negative values $\bar{m}<0$ of a substantial magnitude. This occurs because the majority of offspring will end up in an environment opposite to that of their parents. Although this simple example thus suggests that our findings extend to spatial contexts, more work is needed to assess how maternal effects evolve in more complicated, spatio‐temporal environments.

Another assumption is that maternal effects *m* are expressed by offspring, rather than by the mother. However, additional simulations show that outcomes do not depend on maternal versus offspring expression of *m* (results not shown). This is unsurprising, as offspring fitness is independent of that of its siblings, so that parent–offspring conflict is absent. It would be interesting to relax this assumption in future studies, for example by modeling maternal effects in viscous populations where relatives interact (Uller and Pen, 2011; Kuijper and Johnstone, 2012). Alternatively, one could model the evolution of maternal effects *m* when the phenotype *z* reflects offspring size, which trades‐off with maternal fecundity as in classical life‐history theory (Smith and Fretwell, 1974; Parker and Macnair, 1978; Parker and Begon, 1986). Preliminary results of the latter scenario show that offspring size $z_{t}$ indeed diverges between mother and offspring, as expected. However, the difference in offspring size is entirely caused by differences in the evolved values of the elevation *a*, while values of *m* only attain small values, mirroring our findings for weak selection (Fig. 4B). Values of *m* are small, as survival in classical size‐fecundity models increases monotonically with size (Smith and Fretwell, 1974; Parker and Macnair, 1978), resulting in an open‐ended distribution of surviving maternal phenotypes. As a result, a mother's size is always less informative about the environment relative to a scenario of stabilizing selection in which the distribution of phenotypes is narrowly concentrated around an optimum. An exception to this rule occurs when *m* is expressed by the mother (denoted by *m*_m_), while the elevation *a* and plasticity *b* are expressed by offspring. Here we find that *m*_m_ evolves to very large magnitudes. This is a result of an arms race, in which offspring evolve ever larger values of their elevation and plasticity as they favor an increased size, whereas *m*_m_ evolves to ever smaller (negative) values, as mothers favor a reduced offspring size. Ultimately, extinction follows, as the phenotypic variance explodes when the mean maternal effect becomes smaller than ${\bar{m}}_{m}<-1$ (Kirkpatrick and Lande, 1989), so that more and more offspring are either too small (zt<z min ) or no offspring are produced at all (when $z_{t}=\infty$).

Although the latter outcome seems to be interesting, it remains doubtful whether exclusive maternal expression of *m* is biologically relevant. If *m*_m_ reflects, for example, a manipulative maternal hormone that reduces offspring resource demand, the previously studied scenario implies that offspring can only respond (over evolutionary time) by increasing their expression levels of other substances (through the elevation *a* and plasticity *b*) to compensate for their decrease in demand. Yet, a scenario that is widely considered to be more likely is that offspring are selected to reduce their level of sensitivity to the maternal hormone *m*_m_ in the first place (Müller et al., 2007; Tobler and Smith, 2010) (e.g., by reducing the number of hormone receptor binding sites, Groothuis and Schwabl, 2008). In that case, the evolved value of the maternal effect *m* will be the result of a combined interaction between gene loci expressed in mother and offspring, rather than a result of maternal loci alone. In the context of dispersal, a previous model by Uller and Pen (2011) has demonstrated that the evolution of offspring insensitivity to maternal manipulation generally results in offspring “winning” the conflict, so that the value of maternal effects reflects the offspring's optimum, rather than that of the mother. Hence, assuming that offspring express *m* (rather than their mothers) is likely to be a more reasonable choice when making predictions regarding the strength and magnitude of maternal effects in the long term.

## Supporting information


**Figure S1**: Individual‐based simulations of populations that endure a rapid environmental shift exhibit evolutionary dynamics that are similar to those of the analytical model in Figure 2, at least with respect to characters ${\bar{a}}_{t}$ and ${\bar{m}}_{t}$.
**Figure S2**: Numerical iterations showing adaptation to a sudden shift in the environment, similar to Figure 2, except that the amount of additive genetic variance in maternal effects is larger ($G_{mm}=0.045$ instead of $G_{mm}=0.005$) which increases the phenotypic variance (equation [10]).
**Figure S3**: Numerical iterations of the evolution of the mean maternal effect ${\bar{m}}_{t}$ in response to different magnitudes δ of the environmental shift, while varying the cost of the maternal effect $\omega_{m}^{-2}$.
**Figure S4**: Numerical iterations showing adaptation to more gradual shifts in the environment ${ɛ}_{t}$ for different populations that vary in the presence or absence of within‐generational plasticity, $b_{t}$.
**Figure S5**: Individual‐based simulations showing adaptation to a stochastic temporally fluctuating environment when selection is strong ($\omega_{z}^{2}=0.7$).
**Figure S6**: Individual‐based simulations depicting the evolution of maternal effects ${\bar{m}}_{t}$ (in the absence of plasticity) in a spatial environment.

## MEAN FITNESS

From equation (2) , we can calculate mean fitness $\bar{W}$ by calculating the integral

(A1) $$\bar{W}=\int \;\;\int \;\;\int_{-\infty }^{\infty }W\left(z_{t},b_{t},m_{t}\right)p\left(z_{t},b_{t},m_{t}\right)dz_{t}db_{t}dm_{t},$$

where $p(z_{t},b_{t},m_{t})$ is a trivariate Gaussian distribution with variance–covariance matrix

$$C=\left[\begin{array}{ccc} \sigma_{z_{t}}^{2} & C_{z_{t}b_{t}} & C_{z_{t}m_{t}}\\ C_{z_{t}b_{t}} & G_{bb} & 0\\ C_{z_{t}m_{t}} & 0 & G_{mm} \end{array}\right].$$

Covariances between maternal effects and plasticity are assumed to be absent, as we assume $G_{mb}=0$. The other covariances are not necessarily 0, as effects of phenotypic plasticity and maternal effects on phenotype may generate covariances.

## AVERAGE PHENOTYPES

Taking the expectation of equation (1), we have

(A2) $$\begin{array}{ccc} {\bar{z}}_{t} & = & {\bar{a}}_{t}+{\bar{b}}_{t}{ɛ}_{t-\tau }+\bar{m_{t}z_{t-1}^{*}}\\ & = & {\bar{a}}_{t}+{\bar{b}}_{t}{ɛ}_{t-\tau }+{\bar{m}}_{t}{\bar{z}}_{t-1}^{*}+C_{m_{t}z_{t-1}^{*}}, \end{array}$$

where $C_{m_{t}z_{t-1}^{*}}$ is the covariance between the maternal effect and the maternal phenotype after selection. Subsequently, we assess how $C_{m_{t}z_{t-1}^{*}}$ and ${\bar{z}}_{t-1}^{*}$ depend on ${\bar{a}}_{t}$, ${\bar{b}}_{t}$, and ${\bar{m}}_{t}$. First, we calculate $E_{t-1}\left[z_{t-1}^{*}\right]$, yielding

(A3) $$\begin{array}{ccc} {\bar{z}}_{t-1}^{*} & = & {\bar{a}}_{t-1}^{*}+{\bar{b}}_{t-1}^{*}{ɛ}_{t-\tau -1}+C_{m_{t-1}^{*}z_{t-2}^{*}}+{\bar{m}}_{t-1}^{*}{\bar{z}}_{t-2}^{*},\\ & = & {\bar{a}}_{t}+{\bar{b}}_{t}{ɛ}_{t-\tau -1}+C_{m_{t-1}^{*}z_{t-2}^{*}}+{\bar{m}}_{t}{\bar{z}}_{t-2}^{*}, \end{array}$$

where we assume that breeding values for *a*, *b*, and *m* are transmitted without bias from parents to offspring (implying weak selection and random mating; Falconer 1985; Hadfield 2012), so that ${\bar{a}}_{t-1}^{*}\approx {\bar{a}}_{t}$, ${\bar{b}}_{t-1}^{*}\approx {\bar{b}}_{t}$, and ${\bar{m}}_{t-1}^{*}\approx {\bar{m}}_{t}$. Moreover, note that neither $C_{m_{t-1}^{*}z_{t-2}^{*}}$ nor ${\bar{z}}_{t-2}^{*}$ depend on ${\bar{a}}_{t}$, ${\bar{b}}_{t}$, or ${\bar{m}}_{t}$.

Next, we work out the covariance $C_{m_{t}z_{t-1}^{*}}$ in equation (A2). Starting from the expression of an individual parental phenotype after selection

$$z_{t-1}^{*}=a_{t-1}^{*}+b_{t-1}^{*}{ɛ}_{t-\tau -1}+m_{t-1}^{*}z_{t-2}^{*}+e_{t-1},$$

we have

$$\begin{array}{ccc} C_{m_{t}z_{t-1}^{*}} & = & \bar{m_{t}z_{t-1}^{*}}-{\bar{m}}_{t}{\bar{z}}_{t-1}^{*}\\ & = & \bar{m_{t}\left(a_{t-1}^{*}+b_{t-1}^{*}{ɛ}_{t-\tau -1}+m_{t-1}^{*}z_{t-2}^{*}+e_{t-1}\right)}\\ & - & {\bar{m}}_{t}\bar{\left(a_{t-1}^{*}+b_{t-1}^{*}{ɛ}_{t-\tau -1}+m_{t-1}^{*}z_{t-2}^{*}+e_{t-1}\right)}\\ & = & \bar{m_{t}m_{t-1}^{*}z_{t-2}^{*}}-{\bar{m}}_{t}\bar{m_{t-1}^{*}z_{t-2}^{*}}, \end{array}$$

as $G_{am}=G_{bm}=0$. This can be rewritten

$$\begin{array}{ccc} C_{m_{t}z_{t-1}^{*}} & = & \bar{\left(m_{t}-{\bar{m}}_{t}\right)\left(m_{t-1}^{*}-{\bar{m}}_{t-1}^{*}\right)\left(z_{t-2}^{*}-{\bar{z}}_{t-2}^{*}\right)}\\ & & +{\bar{m}}_{t-1}^{*}C_{m_{t}z_{t-2}^{*}}+{\bar{z}}_{t-2}^{*}C_{m_{t}m_{t-1}^{*}}. \end{array}$$

As third‐order central moments vanish for normally distributed variables, i.e., $E[\left(x-\bar{x}\right)\left(y-\bar{y}\right)\left(z-\bar{z}\right)]=0$, we have

(A4) $$\begin{array}{ccc} C_{m_{t}z_{t-1}^{*}} & = & {\bar{m}}_{t-1}^{*}C_{m_{t}z_{t-2}^{*}}+{\bar{z}}_{t-2}^{*}C_{m_{t}m_{t-1}^{*}}\\ & = & {\bar{m}}_{t}C_{m_{t}z_{t-2}^{*}}+\frac{1}{2}{\bar{z}}_{t-2}^{*}G_{mm}, \end{array}$$

where we make the approximation (assuming weak selection and trait values close to equilibrium) $C_{m_{t}m_{t-1}^{*}}\approx \frac{1}{2}G_{mm}$. Substituting (A3, A4) back into (A2) then yields

(A5) $$\begin{array}{ccc} {\bar{z}}_{t}\; & = & \;\left(1+{\bar{m}}_{t}\right){\bar{a}}_{t}+{\bar{b}}_{t}{ɛ}_{t-\tau }+{\bar{m}}_{t}{\bar{b}}_{t}{ɛ}_{t-\tau -1}\\ & + & {\bar{m}}_{t}\left(C_{m_{t-1}^{*}z_{t-2}^{*}}\;+\;{\bar{m}}_{t}{\bar{z}}_{t-2}^{*}\right)\;+\;{\bar{m}}_{t}C_{m_{t}z_{t-2}^{*}}\;+\;\frac{1}{2}{\bar{z}}_{t-2}^{*}G_{mm}. \end{array}$$

## PHENOTYPIC VARIANCE

Here we derive an expression for the phenotypic variance $\sigma_{z_{t}}^{2}$ at time *t* to work out the derivatives of  ln W¯t. Calculating the variance from equation (1), we have the following expression for the phenotype variance $\sigma_{z_{t}}^{2}$,

(A6) $$\begin{array}{ccc} \sigma_{z_{t}}^{2} & = & G_{aa}+G_{bb}{ɛ}_{t-\tau }^{2}+\sigma_{e}^{2}+2\bar{\left(a_{t}-{\bar{a}}_{t}\right)\left(m_{t}z_{t-1}^{*}-\bar{m_{t}z_{t-1}^{*}}\right)}\\ & + & 2{ɛ}_{t-\tau }\bar{\left(b_{t}-{\bar{b}}_{t}\right)\left(m_{t}z_{t-1}^{*}-\bar{m_{t}z_{t-1}^{*}}\right)}+\bar{{\left(m_{t}z_{t-1}^{*}-\bar{m_{t}z_{t-1}^{*}}\right)}^{2}}, \end{array}$$

where

(A7) $$\begin{array}{ccc} \bar{\left(a_{t}-{\bar{a}}_{t}\right)\left(m_{t}z_{t-1}^{*}-\bar{m_{t}z_{t-1}^{*}}\right)} & = & \bar{\left(a_{t}-{\bar{a}}_{t}\right)\left(m_{t}-{\bar{m}}_{t}\right)\left(z_{t-1}^{*}-\bar{z_{t-1}^{*}}\right)}\\ & + & {\bar{m}}_{t}\bar{a_{t}z_{t-1}^{*}}+{\bar{z}}_{t-1}^{*}\bar{a_{t}m_{t}}-2{\bar{a}}_{t}{\bar{m}}_{t}{\bar{z}}_{t-1}^{*}, \end{array}$$

(A8) $$={\bar{m}}_{t}C_{a_{t}z_{t-1}^{*}}+{\bar{z}}_{t-1}^{*}G_{am}={\bar{m}}_{t}C_{a_{t}z_{t-1}^{*}},$$

again as we assume that $m_{t}$, $a_{t}$, and $z_{t-1}^{*}$ are multivariate normal and the third‐order central moment is zero. Multivariate normality is warranted when trait values $a_{t}$ and $m_{t}$ are the result of a large number of loci of small effect and phenotypic selection is weak.

Similarly,

(A9) $$\bar{\left(b_{t}-{\bar{b}}_{t}\right)\left(m_{t}z_{t-1}^{*}-\bar{m_{t}z_{t-1}^{*}}\right)}={\bar{m}}_{t}C_{b_{t}z_{t-1}^{*}}.$$

Furthermore, we have

$$\begin{array}{ccc} \bar{{\left(m_{t}z_{t-1}^{*}-\bar{m_{t}z_{t-1}^{*}}\right)}^{2}} & = & \bar{m_{t}^{2}{\left(z_{t-1}^{*}\right)}^{2}}-{\left(\bar{m_{t}z_{t-1}^{*}}\right)}^{2}\\ & = & \bar{m_{t}^{2}{\left(z_{t-1}^{*}\right)}^{2}}-{\left(C_{m_{t}z_{t-1}^{*}}+{\bar{m}}_{t}{\bar{z}}_{t-1}^{*}\right)}^{2}. \end{array}$$

This can be further simplified by noting that the fourth‐order central moment satisfies the identity

(A10) $$\bar{{\left(m_{t}-{\bar{m}}_{t}\right)}^{2}{\left(z_{t-1}^{*}-{\bar{z}}_{t-1}^{*}\right)}^{2}}=G_{mm}\sigma_{z_{t-1}^{*}}^{2}+2C_{m_{t}z_{t-1}^{*}}^{2},$$

as E[(x−x¯)2(y−y¯)2]= var (x) var (y)+2 cov (x,y)2 in case of multivariate normality. Expanding the left‐hand side of (A10) gives after quite some algebra,

$$\begin{array}{ccc} \bar{m_{t}^{2}{\left(z_{t-1}^{*}\right)}^{2}} & - & 4C_{m_{t}z_{t-1}^{*}}{\bar{m}}_{t}{\bar{z}}_{t-1}^{*}-G_{mm}{\left({\bar{z}}_{t-1}^{*}\right)}^{2}-{\bar{m}}_{t}^{2}\sigma_{z_{t-1}^{*}}^{2}\\ & & -{\bar{m}}_{t}^{2}{\left({\bar{z}}_{t-1}^{*}\right)}^{2}=G_{mm}\sigma_{z_{t-1}^{*}}^{2}+2C_{m_{t}z_{t-1}^{*}}^{2}, \end{array}$$

and so

$$\begin{array}{ccc} \bar{{\left(m_{t}z_{t-1}^{*}-\bar{m_{t}z_{t-1}^{*}}\right)}^{2}} & = & \left(G_{mm}+{\bar{m}}_{t}^{2}\right)\sigma_{z_{t-1}^{*}}^{2}+C_{m_{t}z_{t-1}^{*}}^{2}\\ & + & 2C_{m_{t}z_{t-1}}{\bar{m}}_{t}{\bar{z}}_{t-1}^{*}+G_{mm}{\left({\bar{z}}_{t-1}^{*}\right)}^{2}. \end{array}$$

Substituting all this into the expression for $\sigma_{z_{t}}^{2}$ (A6) gives

(A11) $$\begin{array}{ccc} \sigma_{z_{t}}^{2} & = & G_{aa}+G_{bb}{ɛ}_{t-\tau }^{2}+\sigma_{e}^{2}+2{\bar{m}}_{t}\left(C_{a_{t}z_{t-1}^{*}}+{ɛ}_{t-\tau }C_{b_{t}z_{t-1}^{*}}\right)\\ & & +\left(G_{mm}+{\bar{m}}_{t}^{2}\right)\sigma_{z_{t-1}^{*}}^{2}+C_{m_{t}z_{t-1}^{*}}^{2}+2C_{m_{t}z_{t-1}^{*}}{\bar{m}}_{t}{\bar{z}}_{t-1}^{*}\\ & & +\;G_{mm}{\left({\bar{z}}_{t-1}^{*}\right)}^{2}. \end{array}$$

## DERIVATIVES OF ${\bar{z}}_{t}$ AND σ^2^

Taking the derivatives of equation (A5) with respect to ${\bar{a}}_{t}$, ${\bar{b}}_{t}$, and ${\bar{m}}_{t}$, we have

(A12a) $$\frac{\partial {\bar{z}}_{t}}{\partial {\bar{a}}_{t}}=1+{\bar{m}}_{t}$$

(A12b) $$\frac{\partial {\bar{z}}_{t}}{\partial {\bar{b}}_{t}}={ɛ}_{t-\tau }+{\bar{m}}_{t}{ɛ}_{t-\tau -1}$$

(A12c) $$\begin{array}{cc} \;\frac{\partial {\bar{z}}_{t}}{\partial {\bar{m}}_{t}} & ={\bar{z}}_{t-1}^{*}+C_{m_{t}z_{t-2}^{*}}+{\bar{m}}_{t}{\bar{z}}_{t-2}^{*}\\ & \approx {\bar{z}}_{t-1}^{*}+\frac{1}{2}C_{m_{t}z_{t-1}^{*}}+{\bar{m}}_{t}{\bar{z}}_{t-2}^{*}, \end{array}$$

where we approximate $C_{m_{t}z_{t-2}^{*}}$ with $\frac{1}{2}C_{m_{t}z_{t-1}^{*}}$. When doing the same for the corresponding derivatives of $\sigma_{z_{t-1}}^{2}$ at time *t*, we note that the phenotypic variance in equation (A11) depends on $\sigma_{z_{t-1}^{*}}^{2}$, which in turn depends on $\sigma_{z_{t-2}^{*}}^{2}$ and so on. To make further progress, we assume that the phenotypic variances change slowly over time and approximate $\sigma_{z_{t-1}}^{2}\approx \sigma_{z_{t-1}^{*}}^{2}$ giving

(A13) $$\begin{array}{ccc} & & \left(1-G_{mm}-{\bar{m}}_{t}^{2}\right)\sigma_{z_{t}}^{2}=G_{aa}+G_{bb}{ɛ}_{t-\tau }^{2}+\sigma_{e}^{2}+2{\bar{m}}_{t}\left(C_{a_{t}z_{t-1}^{*}}\right.\\ & & \left.+{ɛ}_{t-\tau }C_{b_{t}z_{t-1}^{*}}\right)+C_{m_{t}z_{t-1}^{*}}^{2}+2C_{m_{t}z_{t-1}^{*}}{\bar{m}}_{t}{\bar{z}}_{t-1}^{*}+G_{mm}{\left({\bar{z}}_{t-1}^{*}\right)}^{2}. \end{array}$$

Under the close‐to‐equilibrium, weak selection assumption we find

(A14) $$\begin{array}{ccc} C_{a_{t}z_{t-1}^{*}} & = & C_{a_{t}a_{t-1}^{*}}+{\bar{m}}_{t-1}^{*}C_{a_{t}z_{t-2}^{*}}\\ & & \approx \frac{1}{2}G_{aa}+{\bar{m}}_{t}C_{a_{t}z_{t-2}^{*}}, \end{array}$$

(A15) $$\begin{array}{ccc} C_{b_{t}z_{t-1}^{*}} & = & C_{b_{t}b_{t-1}^{*}}{ɛ}_{t-\tau -1}+{\bar{m}}_{t-1}^{*}C_{b_{t}z_{t-2}^{*}}\\ & & \approx \frac{1}{2}G_{bb}{ɛ}_{t-\tau -1}+{\bar{m}}_{t}C_{b_{t}z_{t-2}^{*}}. \end{array}$$

Using these together with equations (A3) and (A4) and the approximations

(A16) $$\begin{array}{c} C_{a_{t}z_{t-2}^{*}}\approx \frac{1}{2}C_{a_{t}z_{t-1}^{*}},\;C_{b_{t}z_{t-2}^{*}}\approx \frac{1}{2}C_{b_{t}z_{t-1}^{*}}\;\text{and}\;C_{m_{t}z_{t-2}^{*}}=\frac{1}{2}C_{m_{t}z_{t-1}^{*}} \end{array}$$

yields

(A17a) $$\frac{\partial \sigma_{z_{t}}^{2}}{\partial {\bar{a}}_{t}}\approx \frac{2}{1-G_{mm}-{\bar{m}}_{t}^{2}}\left(C_{m_{t}z_{t-1}^{*}}{\bar{m}}_{t}+G_{mm}{\bar{z}}_{t-1}^{*}\right)$$

(A17b) $$\frac{\partial \sigma_{z_{t}}^{2}}{\partial {\bar{b}}_{t}}\approx \frac{2{ɛ}_{t-\tau -1}}{1-G_{mm}-{\bar{m}}_{t}^{2}}\left(C_{m_{t}z_{t-1}^{*}}{\bar{m}}_{t}+G_{mm}{\bar{z}}_{t-1}^{*}\right)$$

(A17c) $$\begin{array}{ccc} \frac{\partial \sigma_{z_{t}}^{2}}{\partial {\bar{m}}_{t}}\; & \approx & \frac{2}{1-G_{mm}-{\bar{m}}_{t}^{2}}(\left[1+\frac{1}{2}{\bar{m}}_{t}\right]C_{a_{t}z_{t-1}^{*}}\;+\;\left[{ɛ}_{t-\tau }\;+\;\frac{1}{2}{\bar{m}}_{t}{ɛ}_{t-\tau -1}\right]\\ & \times & \;C_{b_{t}z_{t-1}^{*}}+C_{m_{t}z_{t-1}^{*}}\left[\frac{1}{2}C_{m_{t}z_{t-1}^{*}}+{\bar{z}}_{t-1}^{*}\left(1+\frac{1}{2}{\bar{m}}_{t}\right)+{\bar{m}}_{t}{\bar{z}}_{t-2}^{*}\right]\\ & & +\;G_{mm}{\bar{z}}_{t-1}^{*}{\bar{z}}_{t-2}^{*}+{\bar{m}}_{t}\sigma_{z_{t}}^{2}). \end{array}$$

## UPDATE RULES FOR ${\bar{z}}_{t}^{*}$ AND COVARIANCES

To update the phenotypic components each time step, we also need to update ${\bar{z}}_{t}^{*}$. Referring to equation (A3) gives

(A18) $${\bar{z}}_{t}^{*}={\bar{a}}_{t+1}+{\bar{b}}_{t+1}{ɛ}_{t-\tau }+C_{m_{t}^{*}z_{t-1}^{*}}+{\bar{m}}_{t+1}{\bar{z}}_{t-1}^{*}.$$

To make further progress we approximate $C_{m_{t}^{*}z_{t-1}^{*}}\approx C_{m_{t}z_{t-1}^{*}}$, and so

(A19) $${\bar{z}}_{t}^{*}\approx {\bar{a}}_{t+1}+{\bar{b}}_{t+1}{ɛ}_{t-\tau }+C_{m_{t}z_{t-1}^{*}}+{\bar{m}}_{t+1}{\bar{z}}_{t-1}^{*}.$$

To step forward in time for a given sequence of environments, we need to find $C_{m_{t+1}z_{t}^{*}}$, $C_{a_{t+1}z_{t}^{*}}$, and $C_{b_{t+1}z_{t}^{*}}$ in terms of known quantities at time *t*. From equation (A4) we have

$$C_{m_{t+1}z_{t}^{*}}={\bar{m}}_{t+1}C_{m_{t+1}z_{t-1}^{*}}+\frac{1}{2}{\bar{z}}_{t-1}^{*}G_{mm}.$$

Under the weak selection, close to equilibrium assumption we approximate $C_{m_{t+1}z_{t-1}^{*}}\approx \left(1/2\right)C_{m_{t}z_{t-1}^{*}}$ to get

(A20) $$C_{m_{t+1}z_{t}^{*}}\approx \frac{1}{2}{\bar{m}}_{t+1}C_{m_{t}z_{t-1}^{*}}+\frac{1}{2}{\bar{z}}_{t-1}^{*}G_{mm}.$$

From equations (A14) and (A15) we also have

(A21) $$\begin{array}{cc} C_{a_{t+1}z_{t}^{*}} & \approx \frac{1}{2}G_{aa}+{\bar{m}}_{t+1}C_{a_{t+1}z_{t-1}^{*}}\\ & \approx \frac{1}{2}G_{aa}+\frac{1}{2}{\bar{m}}_{t+1}C_{a_{t}z_{t-1}^{*}}, \end{array}$$

(A22) $$\begin{array}{cc} C_{b_{t+1}z_{t}^{*}} & \approx \frac{1}{2}G_{bb}{ɛ}_{t-\tau }+{\bar{m}}_{t+1}C_{b_{t+1}z_{t-1}^{*}}\\ & \approx \frac{1}{2}G_{bb}{ɛ}_{t-\tau }+\frac{1}{2}{\bar{m}}_{t+1}C_{b_{t}z_{t-1}^{*}}, \end{array}$$

using the equivalent of approximations (A16).

## EQUILIBRIUM SOLUTIONS IN CONSTANT ENVIRONMENTS

We look for equilibrium solutions to equations (8a)–(8c) in a constant environment ${ɛ}_{t}\equiv ɛ$. Setting $\Delta {\bar{a}}_{t}=0$ in equation (8a) gives

(A23) $$\left(\bar{z}-\theta \right)\frac{\partial {\bar{z}}_{t}}{\partial {\bar{a}}_{t}}=-\frac{1}{2}\frac{\partial \bar{\sigma_{z}^{2}}}{\partial {\bar{a}}_{t}}$$

at a leading order, where at equilibrium, ${\bar{z}}_{t}=\bar{z}$ is constant. Using equations (A12a) and (A17a) and approximating $C_{m_{t}z_{t-1}^{*}}\approx \bar{z}G_{mm}/\left(2-\bar{m}\right)$ at equilibrium from equation (A20), for constant $\bar{m}$, gives

(A24) $$\left(\bar{z}-\theta \right)\left(1+\bar{m}\right)=-\frac{2\bar{z}G_{mm}}{\left(1-G_{mm}-{\bar{m}}^{2}\right)\left(2-\bar{m}\right)}.$$

Similarly we can derive

(A25) $$ɛ\left(\bar{z}-\theta \right)\left(1+\bar{m}\right)=-\frac{2ɛ\bar{z}G_{mm}}{\left(1-G_{mm}-{\bar{m}}^{2}\right)\left(2-\bar{m}\right)}-\frac{\omega_{z}^{2}\bar{b}}{\omega_{b}^{2}},$$

from equations (8b), (A12b), and (A17b). Comparing this to equation (A24) we see that when there are costs of plasticity, all equilibrium solutions have $\bar{b}=0$.

Setting $\Delta {\bar{m}}_{t}=0$ in equation (8c) gives

$$\left(\bar{z}-\theta \right)\frac{\partial {\bar{z}}_{t}}{\partial {\bar{m}}_{t}}=-\frac{1}{2}\frac{\partial \bar{\sigma_{z}^{2}}}{\partial {\bar{m}}_{t}}-\frac{\omega_{z}^{2}\bar{m}}{\omega_{m}^{2}},$$

and using equations (A12c) and (A17c) and approximating

(A26a) $$C_{a_{t}z_{t-1}^{*}}\approx \frac{G_{aa}}{\left(2-\bar{m}\right)},$$

(A26b) $$C_{b_{t}z_{t-1}^{*}}\approx \frac{ɛG_{bb}}{\left(2-\bar{m}\right)},$$

(A26c) $$C_{m_{t}z_{t-1}^{*}}\approx \frac{\bar{z}G_{mm}}{\left(2-\bar{m}\right)}$$

at equilibrium from equations (A21)–(A20) gives

$$\begin{array}{ccc} & & \left[\bar{z}\left(1+\bar{m}\right)+\frac{\bar{z}G_{mm}}{2(2-\bar{m})}\right]\left(\bar{z}-\theta \right)=-\frac{1}{2(1-G_{mm}-{\bar{m}}^{2})}\\ & & \times [\frac{2+\bar{m}}{2-\bar{m}}\left(G_{aa}+{ɛ}^{2}G_{bb}+{\bar{z}}^{2}G_{mm}\right)+\frac{{\bar{z}}^{2}G_{mm}^{2}}{{\left(2-\bar{m}\right)}^{2}}+\frac{2\bar{m}{\bar{z}}^{2}G_{mm}}{2-\bar{m}}\\ & & +2G_{mm}{\bar{z}}^{2}+2\bar{m}\sigma_{z}^{2}]-\frac{\omega_{z}^{2}\bar{m}}{\omega_{m}^{2}}. \end{array}$$

Now substituting for $\left(\bar{z}-\theta \right)$ from equation (A24), rearranging and simplifying gives

(A27) $$\begin{array}{c} \frac{2+\bar{m}}{2-\bar{m}}\left(G_{aa}+{ɛ}^{2}G_{bb}\right)+\frac{{\bar{z}}^{2}G_{mm}f\left(\bar{m}\right)}{{\left(2-\bar{m}\right)}^{2}\left(1+\bar{m}\right)}\\ +\;2\bar{m}\sigma_{z}^{2}+\frac{2\omega_{z}^{2}\bar{m}}{\omega_{m}^{2}}\left(1-G_{mm}-{\bar{m}}^{2}\right)=0, \end{array}$$

where

$$f\left(\bar{m}\right)=\left(-1+\bar{m}\right)G_{mm}+\left(4-{\bar{m}}^{2}\right)\left(1+\bar{m}\right).$$

From equation (A13), using approximations (A26a)–(A26c) we see that at equilibrium, the phenotypic variance is approximately

(A28) $$\begin{array}{ccc} \sigma_{z_{t}}^{2} & & \approx \frac{1}{1-G_{mm}-{\bar{m}}^{2}}\left[\frac{2+\bar{m}}{2-\bar{m}}\left(G_{aa}+G_{bb}{ɛ}^{2}+G_{mm}{\bar{z}}^{2}\right)\right.\\ & & \left.+\;\frac{{\bar{z}}^{2}G_{mm}^{2}}{{\left(2-\bar{m}\right)}^{2}}+\sigma_{e}^{2}\right]. \end{array}$$

We want to consider values of *m* in a range around zero. From the expression above, we see that for equilibrium solutions to be possible, we must have $1-G_{mm}-{\bar{m}}^{2}>0$, and so $0\leq G_{mm}<1$ and the range of $\bar{m}$ is then given by $-\sqrt{1-G_{mm}}<\bar{m}<\sqrt{1-G_{mm}}$. Alternatively we can write $0\leq G_{mm}<1-{\bar{m}}^{2}$. Thus we have

(A29) $$\begin{array}{ccc} f\left(\bar{m}\right)\geq g\left(\bar{m}\right) & & \equiv \left(-1+\bar{m}\right)\left(1-{\bar{m}}^{2}\right)+\left(4-{\bar{m}}^{2}\right)\left(1+\bar{m}\right),\\ & = & \left(1+\bar{m}\right)\left(3+2\bar{m}-2{\bar{m}}^{2}\right). \end{array}$$

The function $g(\bar{m})$ has roots at $\bar{m}=-1,-0.823,1.823$, with $g(\bar{m})>0$ for $-0.823<\bar{m}<1.823$. Hence we also have $f(\bar{m})>0$ for $-0.823<\bar{m}<1.823$. Thus if $\bar{m}$ were positive, in the allowed range $0<\bar{m}<\sqrt{1-G_{mm}}$ then all the terms in equation (A27) would be positive and there would be no equilibrium solution possible. Therefore all equilibrium solutions in the range of validity of our model must have negative mean maternal effect coefficient, i.e., $\bar{m}<0$.
