# Supplementary material for: When to rely on maternal effects and when on phenotypic plasticity?
Source: Evolution. 2015 Apr 10;69(4):950–68. doi: 10.1111/evo.12635 (PMC4975690; doi:10.1111/evo.12635)
Supplement: Supplementary file 1 — Figure S1: Individual‐based simulations of populations that endure a rapid environmental shift exhibit evolutionary dynamics that are similar to those of the analytical model in Figure 2, at least with respect to characters a¯t and m¯t. Figure S2: Numerical iterations showing adaptation to a sudden shift in the environment, similar to Figure 2, except that the amount of additive genetic variance in maternal effects is larger (Gmm=0.045 instead of Gmm=0.005) which increases the phenotypic variance (equation [10]). Figure S3: Numerical iterations of the evolution of the mean maternal effect m¯t in response to different magnitudes δ of the environmental shift, while varying the cost of the maternal effect ωm−2. Figure S4: Numerical iterations showing adaptation to more gradual shifts in the environment ɛt for different populations that vary in the presence or absence of within‐generational plasticity, bt. Figure S5: Individual‐based simulations showing adaptation to a stochastic temporally fluctuating environment when selection is strong (ωz2=0.7). Figure S6: Individual‐based simulations depicting the evolution of maternal effects m¯t (in the absence of plasticity) in a spatial environment. [file EVO-69-950-s001.pdf]

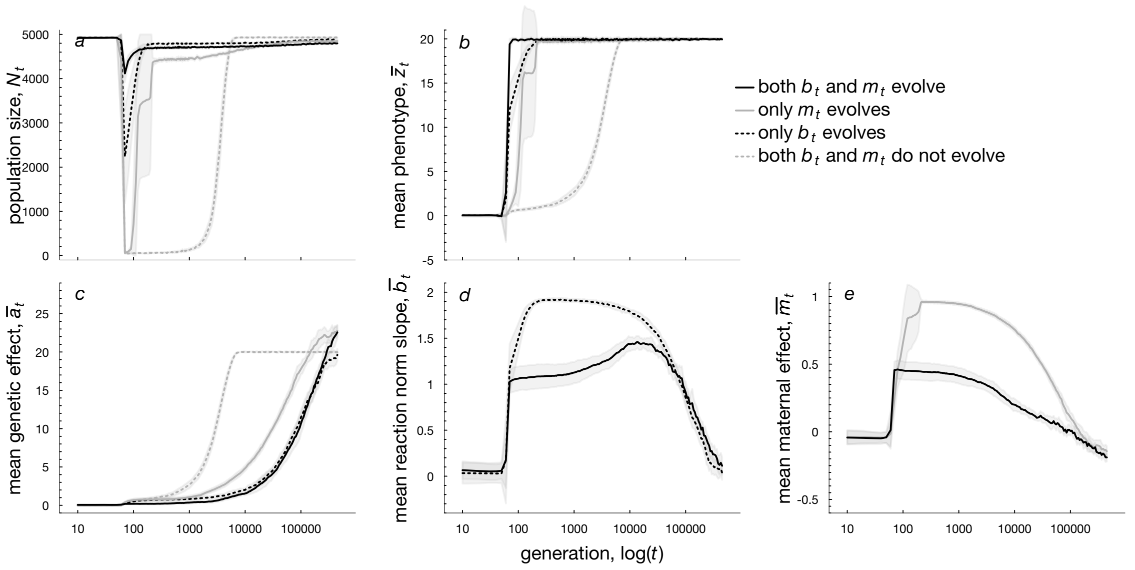

Figure S1:

S1 Supplementary Figures

Figure S1 Individual-based simulations of populations that endure a rapid environmental shift exhibit evolutionary dynamics that are similar to those of the analytical model in Figure 2, at least with respect to characters $\bar{a}_t$ and $\bar{m}_t$. The congruence of both figures indicates that weak-selection assumptions in the analytical model are robust to more realistic situations in which population sizes are finite. Parameters: $N = 5000$, $\mu_a = \mu_b = \mu_m = 0.02$, $\sigma^2_{\mu_b} = \sigma^2_{\mu_m} = \sigma^2_{\mu_z} = 0.0025$, $\omega^2_z = 40$, $\omega^2_b = \omega^2_m = 100$, $\omega^2_{b,\text{high}} = \omega^2_{m,\text{high}} = 1$, $B = 2$, $\sigma^2_\xi = 0.01$, $\rho = 0.5$, $\tau = 0.25$, $\delta = 10$, $\sigma^2_e = 1$. Shaded ranges depict the standard deviations over 10 replicate simulations.

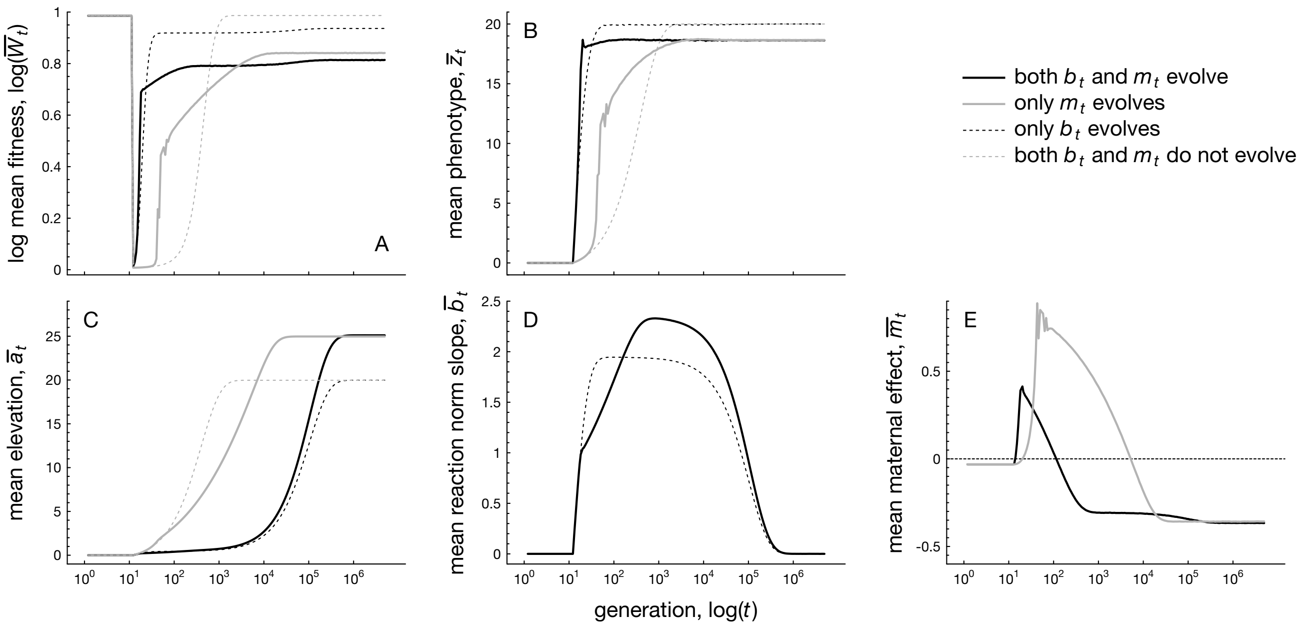

Figure S2:

Figure S2 Numerical iterations showing adaptation to a sudden shift in the environment, similar to Figure 2, except that the amount of additive genetic variance in maternal effects is larger (G_{mm} = 0.045 instead of $G_{mm} = 0.005$) which increases the phenotypic variance (eq. [10]). Minimization of an increased phenotypic variance favors more negative values of $\bar{m}$ in the new environment, which at the same time prevents long-term adaptation to the novel environment in the presence of maternal effects. Parameters: see Figure 2.

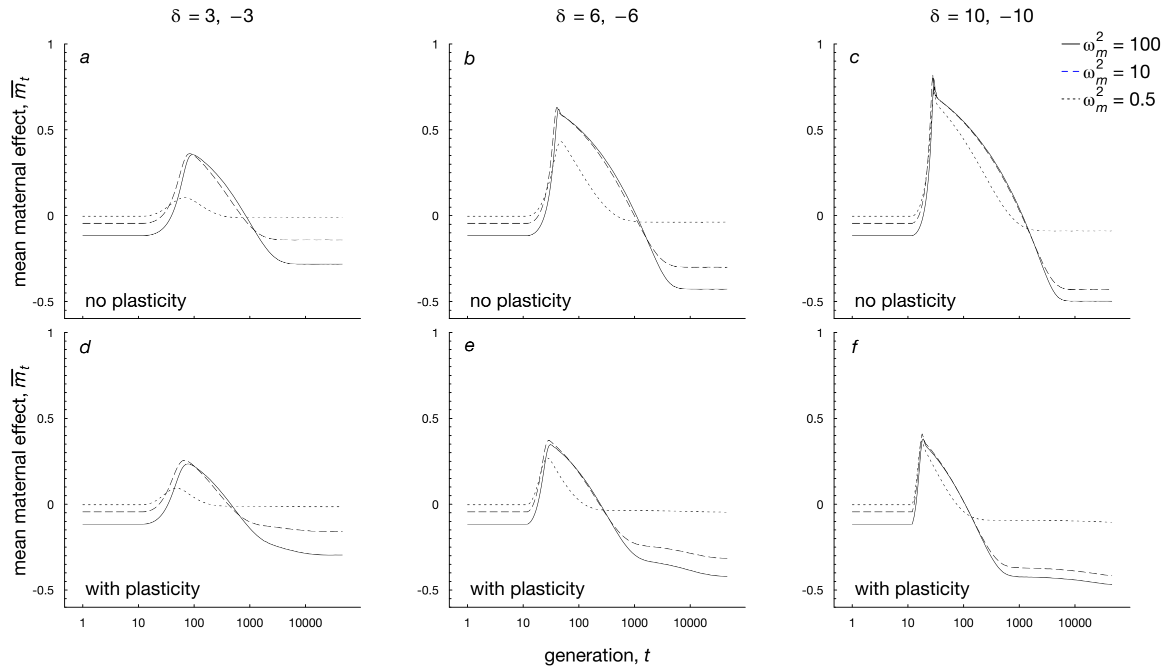

Figure S3:

Figure S3 Numerical iterations of the evolution of the mean maternal effect $\bar{m}_t$ in response to different magnitudes δ of the environmental shift, while varying the cost of the maternal effect ω_m^{-2}. Dotted lines ($\omega_m^2 = 0.5$) reflect that maternal effects are very costly, whereas dashed ($\omega_m^2 =$ 10) and solid lines ($\omega_m^2 = 100$) reflect progressively weaker costs of maternal effects. Panels a-c: in the absence of phenotypic plasticity b_t, maternal effects show a pronounced positive transient response to the environmental shift, even when costs of maternal effects are extremely high, with $\bar{m}$ remaining positive for $>$ 1000 generations. Panels d-f show that this transient response of $\bar{m}_t$ is maintained in the face of phenotypic plasticity (see also Figure 2d,e), although the number of generations during which $\bar{m}$ remains positive is reduced. Parameters: $G_{aa} = 0.1$, $G_{bb} = 0.045$ (panels d-f), $G_{mm} = 0.005$, $\omega_z^2 = 40$, $A = 0$, $B = 2$, $\sigma_\xi^2 = 0.01$, $\rho = 0.5$, $\delta = 10$, $\tau = 0.25$, $\omega_b^2 =$ 100, $\sigma_e^2 = 1$.

Figure S4 Numerical iterations showing adaptation to more gradual shifts in the environment ε_t for different populations that vary in the presence or absence of within-generational plasticity, b_t. The environmental shift initiates at generation $t = 10$ and achieves its new value either after 100, 1000 or 10 000 generations. Panels A, B: evolution of $\bar{z}_t$ and $\bar{m}_t$ when phenotypic plasticity is absent. Panels C-E: evolution of $\bar{z}_t$, $\bar{b}_t$ and $\bar{m}_t$ when phenotypic plasticity is present. Overall, results

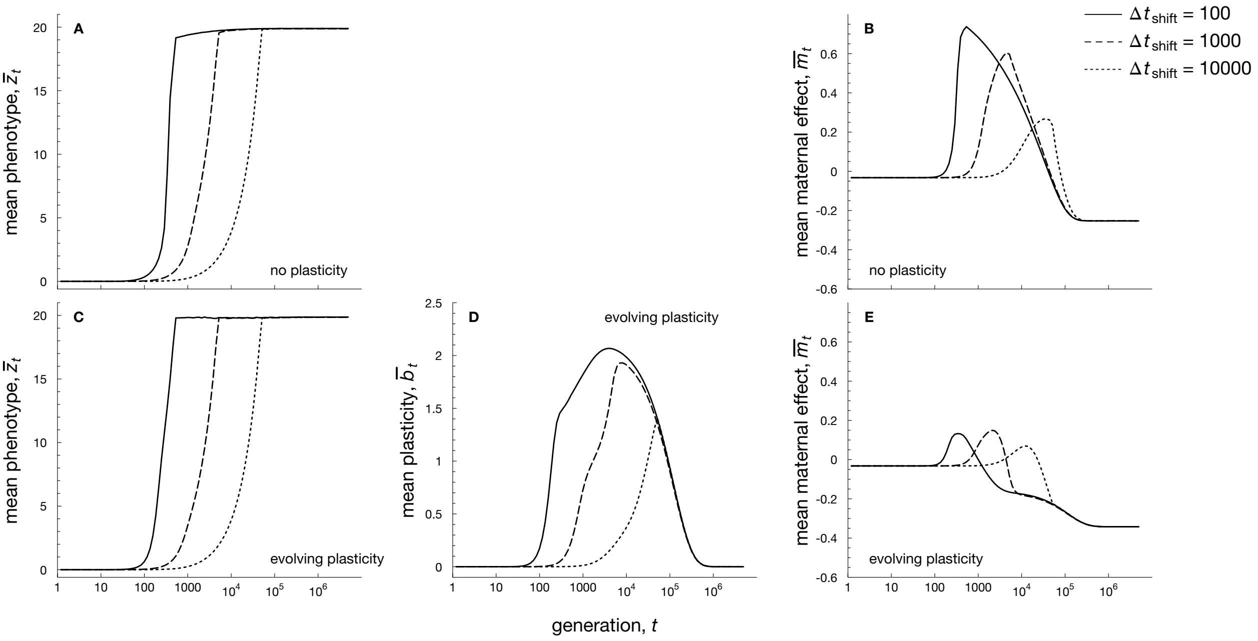

Figure S4:

are highly similar to the sudden environmental shift in Figure 2 that takes place during a single generation. Only when the environmental shift is substantially slow (i.e., 10 000 generations), is gradual change in the elevation $\bar{a}_t$ sufficient to achieve a sufficient response to change, selectively favoring lower values of maternal effects or phenotypic plasticity. Parameters: $G_{aa} = 0.1$, $G_{bb} = 0.045$, $G_{mm} = 0.005$, $\omega_z^2 = 40$, $A = 0$, $B = 2$, $\sigma_\xi^2 = 0.01$, $\rho = 0.5, \delta = 10, \tau = 0.25$, $\sigma_e^2 = 1$.

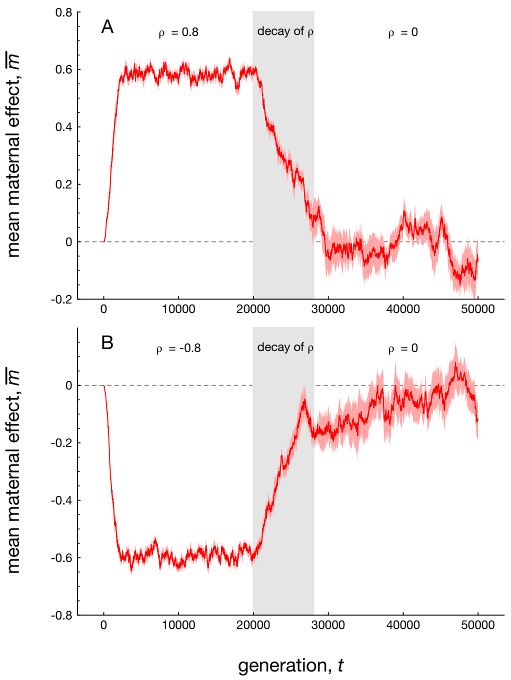

Figure S5:

Figure S5 Individual-based simulations showing adaptation to a stochastic temporally fluctuating environment when selection is strong ($\omega_z^2 = 0.7$). Environmental fluctuations are reflected by the parameter ξ_t in eq. (6), reflecting an autocorrelated Gaussian timeseries with autocorrelation ρ and environmental variance $\sigma_\xi^2 = 1$. During the initial phase of simulation ($0 < t < 20000$) the autocorrelations have a large magnitude, so that $\bar{m}_t$ is selected to be either substantially positive (when $\rho = 0.8$, panel A) or negative (when $\rho = -0.8$, panel B), corroborating findings in a periodic environment (Figure 4d). Between generations $20000 < t < 28000$, autocorrelations gradually decay with a step $\Delta\rho = \pm 0.001$ towards increased unpredictability, leading to a corresponding decrease in the magnitude of $\bar{m}_t$. After $t \geq 28000$, the environment is unpredictable ($\rho = 0$) and values of $\bar{m}_t$ are very slight and, on average, negative. Small values of $\bar{m}_t$ when $\rho = 0$ again reflect findings in the periodic environment where the autocorrelation is absent (e.g., see $f = \frac{1}{2}\pi$ for Figure 4d). Parameters: $\omega_z^2 = 0.7$, $\omega_m^2 = 100$, $B = 2$, $\sigma_\xi^2 = 1.0$, $\tau = 0.25$, $\mu_a = \mu_b = \mu_m = 0.02$, $\sigma_{\mu_b}^2 = \sigma_{\mu_m}^2 = \sigma_{\mu_z}^2 = 0.0025$, $\sigma_e^2 = 1$.

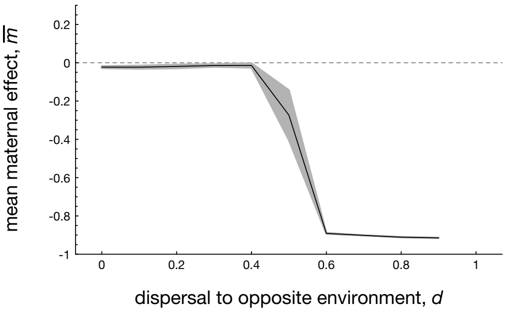

Figure S6:

Figure S6 Individual-based simulations depicting the evolution of maternal effects $\bar{m}_t$ (in the absence of plasticity) in a spatial environment. The environment consists of two patches with respective environmental values $\varepsilon_1 = -1$ and $\varepsilon_2 = 1$. With probability d an offspring disperses to a patch with the opposite environmental value, whereas with probability $1-d$ an offspring remains in the maternal environment. Parameters: $\omega_z^2 = 1.0$, $\omega_m^2 = \omega_b^2 = 100$, $B = 2$, $\sigma_\xi^2 = 0$, $\tau = 0$, $\mu_a = \mu_b = \mu_m = 0.02$, $\sigma_{\mu_b}^2 = \sigma_{\mu_m}^2 = \sigma_{\mu_z}^2 = 0.0025$, $\sigma_e^2 = 1$.
